# Architectures of photosynthetic RC-LH1 supercomplexes from *Rhodobacter blasticus*

**DOI:** 10.1126/sciadv.adp6678

**Published:** 2024-10-09

**Authors:** Peng Wang, Bern M. Christianson, Deniz Ugurlar, Ruichao Mao, Yi Zhang, Ze-Kun Liu, Ying-Yue Zhang, Adrian M. Gardner, Jun Gao, Yu-Zhong Zhang, Lu-Ning Liu

**Affiliations:** ^1^ MOE Key Laboratory of Evolution and Marine Biodiversity, Frontiers Science Center for Deep Ocean Multispheres and Earth System & College of Marine Life Sciences, Ocean University of China, Qingdao 266003, China.; ^2^ Institute of Systems, Molecular and Integrative Biology, University of Liverpool, L69 7ZB Liverpool, United Kingdom.; ^3^ Thermo Fisher Scientific, Life Sciences EMEA, Achtseweg Noord 5, 5651 GG Eindhoven, Netherlands.; ^4^ Hubei Key Laboratory of Agricultural Bioinformatics, College of Informatics, Huazhong Agricultural University, Wuhan 430070, Hubei, China.; ^5^ Department of Chemistry, Stephenson Institute of Renewable Energy, and Early Career Laser Laboratory, University of Liverpool, L69 7ZF Liverpool, UK.

## Abstract

The reaction center–light-harvesting complex 1 (RC-LH1) plays an essential role in the primary reactions of bacterial photosynthesis. Here, we present high-resolution structures of native monomeric and dimeric RC-LH1 supercomplexes from *Rhodobacter* (*Rba.*) *blasticus* using cryo–electron microscopy. The RC-LH1 monomer is composed of an RC encircled by an open LH1 ring comprising 15 αβ heterodimers and a PufX transmembrane polypeptide. In the RC-LH1 dimer, two crossing PufX polypeptides mediate dimerization. Unlike *Rhodabacter sphaeroides* counterpart, *Rba. blasticus* RC-LH1 dimer has a less bent conformation, lacks the PufY subunit near the LH1 opening, and includes two extra LH1 αβ subunits, forming a more enclosed S-shaped LH1 ring. Spectroscopic assays reveal that these unique structural features are accompanied by changes in the kinetics of quinone/quinol trafficking between RC-LH1 and cytochrome *bc*_1_. Our findings reveal the assembly principles and structural variability of photosynthetic RC-LH1 supercomplexes, highlighting diverse strategies used by phototrophic bacteria to optimize light-harvesting and electron transfer in competitive environments.

## Teaser

Structure of a unique RC-LH1 dimer spotlights the structural and functional variability of bacterial photosynthetic systems.

## INTRODUCTION

Photosynthesis is an essential biological process that converts solar energy into chemical energy, which powers almost all life on Earth (*1*). Photosynthetic reaction centers (RCs) are essential pigment-protein complexes of the light-driven electron transport and energy conversion in photosynthesis. In purple photosynthetic bacteria, the membrane-bound RCs closely associate with light-harvesting complex 1 (LH1) to form the RC-LH1 core supercomplexes (*2*–*14*). The LH1 ring of purple bacteria captures photons from solar energy or peripheral antenna complexes, such as LH2, and transports energy to the RC. The RC then uses the energy to initiate a charge separation and electron transfer, ultimately creating proton gradients across the chromatophore membrane to produce adenosine 5′-triphosphate (ATP) (*15*–*17*).

Within the group of anoxygenic purple non-sulfur phototrophs, many species of the *Rhodobacter* (*Rba.*) genus have been recognized as exemplary model organisms for understanding the architecture and function of the natural photosynthetic apparatus. The *Rba.* genus consists of 16 validly recognized species, which exhibit a high degree of variability (*9*, *18*). According to taxogenomic studies, this genus can be categorized into five monophyletic clusters. *Rhodobacter sphaeroides* is a representative of clade I, while *Rhodobacter capsulatus* represents clade II. Clade V is represented by *Rhodobacter vinaykumarii*, while *Rhodobacter blasticus* and *Rhodobacter veldkampii* are the only species found in clade III and clade IV, respectively.

A unique feature of *Rba.* RC-LH1 supercomplexes is the presence of the PufX polypeptide despite their significant diversity in this genus in terms of protein composition, content of pigments and LH1 subunits, and RC-LH1 oligomerization (*10*). Recent cryo–electron microscopy (cryo-EM) studies have revealed the structural details of the RC-LH1 supercomplexes in *Rba. sphaeroides* (*4*–*7*, *12*), *Rba. veldkampii* (*3*), and *Rba. capsulatus* (*8*, *9*). *Rba. sphaeroides* has a dimeric RC-LH1 structure consisting of two RCs and an S-shaped LH1 ring, and the PufX subunit plays a critical role in the dimerization of *Rba. sphaeroides* RC-LH1. In addition to PufX, *Rba. sphaeroides* RC-LH1 contains a distinct subunit, known as PufY (or protein Y), which is thought to regulate the number of αβ subunits in the LH1 ring and stabilize the RC-LH1 dimer (*3*–*7*, *12*, *19*, *20*). In contrast, although having PufX, the RC-LH1 complexes of *Rba. capsulatus* and *Rba. veldkampii* exhibit exclusively monomeric structures (*3*, *8*, *9*). *Rba. blasticus* represents an independent clade within the *Rba.* genus, and it has been suggested that *Rba. blasticus* produces dimeric RC-LH1, similar to that of *Rba. sphaeroides* (*21*). However, the structural and functional details of the *Rba. blasticus* RC-LH1 core complexes have not yet been documented.

Here, we report the cryo-EM structures of the RC-LH1 supercomplexes from *Rba. blasticus*. The high-resolution structures reveal unique architectural features of the PufX-containing RC-LH1 monomers and dimers compared to those of *Rba. sphaeroides*. Structural analysis, together with spectroscopic assays and molecular dynamics (MD) simulations, provides valuable insights into the assembly and electron transport mechanisms of the RC-LH1 complexes. Our study highlights the structural variations and modularity of bacterial photosynthetic core complexes for functional regulation, which play a crucial role in regulating their functionality. An advanced understanding of bacterial photosynthesis could inform redesign and remodeling of light harvesting and electron flow in clean energy biotechnology.

## RESULTS AND DISCUSSION

### Overall structures of the RC-LH1 supercomplexes

Wild-type (WT) *Rba. blasticus* have lamellar-type intracytoplasmic photosynthetic membranes (fig. S1A). We isolated the RC-LH1 supercomplexes from the photosynthetic membranes of cells grown phototrophically under anoxic conditions using sucrose density gradient centrifugation. The results indicate that the lamellar photosynthetic membranes accommodate both RC-LH1 monomers and dimers (fig. S1B), consistent with the observation of their assembly status in photosynthetic membranes using atomic force microscopy (*21*). We then subjected the RC-LH1 monomers and dimers for single-particle cryo-EM analysis.

**Fig. 1.**
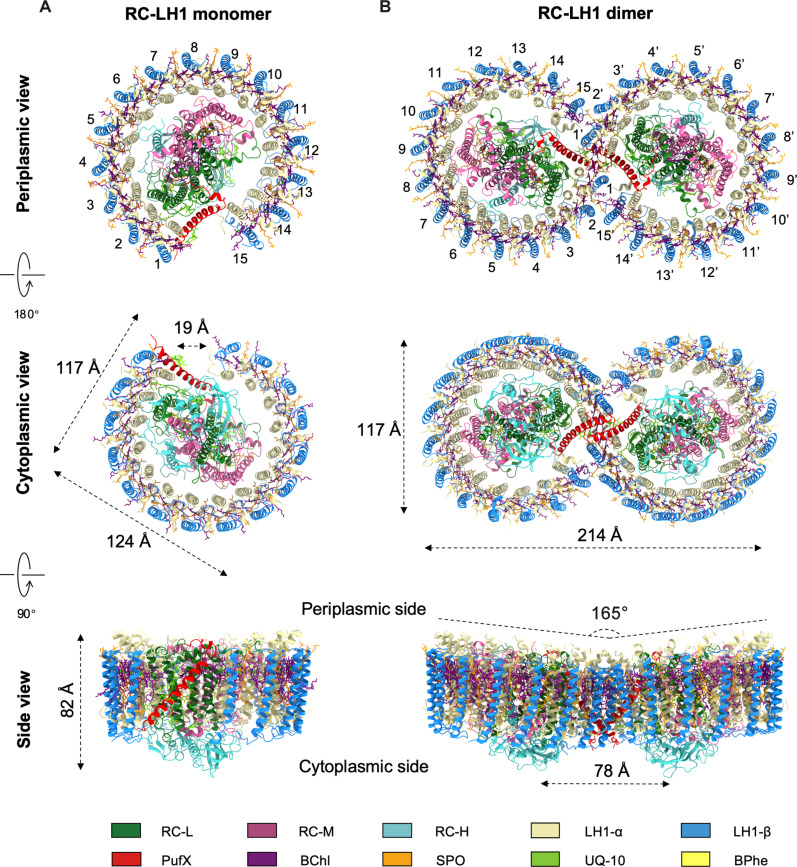
Cryo-EM structures of the RC-LH1 supercomplexes from *Rba. blasticus*. (**A**) RC-LH1 monomer. Top: View from the periplasmic side; middle: view from the cytoplasmic side; bottom: side view in the membrane plane (facing the PufX-mediated gap region). (**B**) RC-LH1 dimer. Top: View from the periplasmic side; middle: view from the cytoplasmic side; bottom: side view in the membrane plane. Color scheme is presented at the bottom as follows: LH1-α, wheat; LH1-β, light blue; PufX, red; RC-L, green; RC-M, magenta; RC-H, light sea green; bacteriochlorophylls (BChls), purple; bacteriopheophytins (BPhes), yellow; carotenoids, orange; quinones, lawn green.

The cryo-EM structure of the WT RC-LH1 monomer at 2.60-Å resolution reveals that the RC-LH1 monomer is composed of 34 protein polypeptides and 87 cofactors, with dimensions of 124 × 117 × 82 Å and a molecular mass of 361 kDa (Fig. 1A and figs. S2 to S4). The RC contains H, L, and M subunits that are almost identical in structure to those in the RCs of other *Rba.* strains (figs. S5 and S6). The RC is encompassed by an open LH1 ring consisting of 15 αβ subunits, with a gap (~19 Å) formed by a tilted PufX transmembrane polypeptide relative to the membrane plane (Fig. 1A and fig. S2A). Differing from the *Rba. sphaeroides* counterpart, the RC-LH1 monomer of *Rba. blasticus* does not contain the PufY subunit (also named as protein Y) and has an additional LH1 subunit, which is structurally similar to the RC-LH1 monomers of *Rba. veldkampii* and *Rba. capsulatus* (fig. S6).

The cryo-EM structure of the WT RC-LH1 dimer at 2.84-Å resolution shows that the RC-LH1 dimer comprises 68 polypeptides and 174 cofactors, adopting a twofold symmetry composed of two identical RC-LH1 monomers (Fig. 1B and figs. S2B, S7, and S8). The long and short dimensions of the dimeric supercomplex are 214 and 117 Å, respectively (Fig. 1B). Within the dimer, two RCs are surrounded by 30 LH1 αβ polypeptides, which form a relatively enclosed S-shaped architecture with two adjacent C-shaped LH1 rings (Fig. 1B and fig. S2B). Two PufX polypeptides are located at the dimerization interface near the two LH1 openings in the center of the RC-LH1 dimer (Fig. 1B and fig. S2B).

The LH1-15 αβ heterodimer exhibits relatively weak local density in the RC-LH1 monomer, which is likely attributed to its relatively low stability within native monomeric RC-LH1. The model was built to indicate only its presence. In contrast, LH1-15 in the WT RC-LH1 dimer appears to be more stable, resulting in a better local density (figs. S2, S3E, and S7E). This difference in density quality suggests that the dimeric form provides a more stable environment for the LH1-15 subunit than the monomeric form.

A striking feature is that the *Rba. blasticus* RC-LH1 dimer is flatter than the *Rba. sphaeroides* counterpart, with a tilted angle of 165° between the two monomers within the dimer in contrast to 152° for the *Rba. sphaeroides* RC-LH1 dimer, similar to the RC-LH1 dimer of *Rhodobaca* (*Rca.*) *bogoriensis* (166°) (Fig. 1B and figs. S2B and S9) (*22*, *23*). All-atom MD (AAMD) simulations revealed that the intrinsically bent architecture of the *Rba. blasticus* RC-LH1 dimer could not create remarkable local membrane curvature (fig. S10). Moreover, the LH1 array of the *Rba. blasticus* RC-LH1 dimer contains additional two LH1 αβ-polypeptides (LH1-15 and LH1-15′), thereby exhibiting a more enclosed “S” shape compared to that of the *Rba. sphaeroides* counterpart (Fig. 1B and figs. S2B and S9). No PufY peptides were detected in the *Rba. blasticus* RC-LH1 dimer, distinct from their counterparts in *Rba. sphaeroides* and *Rca. bogoriensis* (Fig. 1 and figs. S2 and S9) (*4*, *22*, *23*). The gene encoding PufY or its homologs was also not present in the *Rba. blasticus* genome (*24*). In the absence of PufY at the LH1 gate, LH1-13 and LH1-14 shift toward the RC and align with the contour of the RC, and an additional LH1-15 appears. These features are in line with those of the RC-LH1-PufX monomers from *Rba. veldkampii* and *Rba. capsulatus*, as well as the RC-LH1-PufX dimer of *Rca. bogoriensis*.

### Structural basis for the dimerization of *Rba. blasticus* RC-LH1

**Fig. 2.**
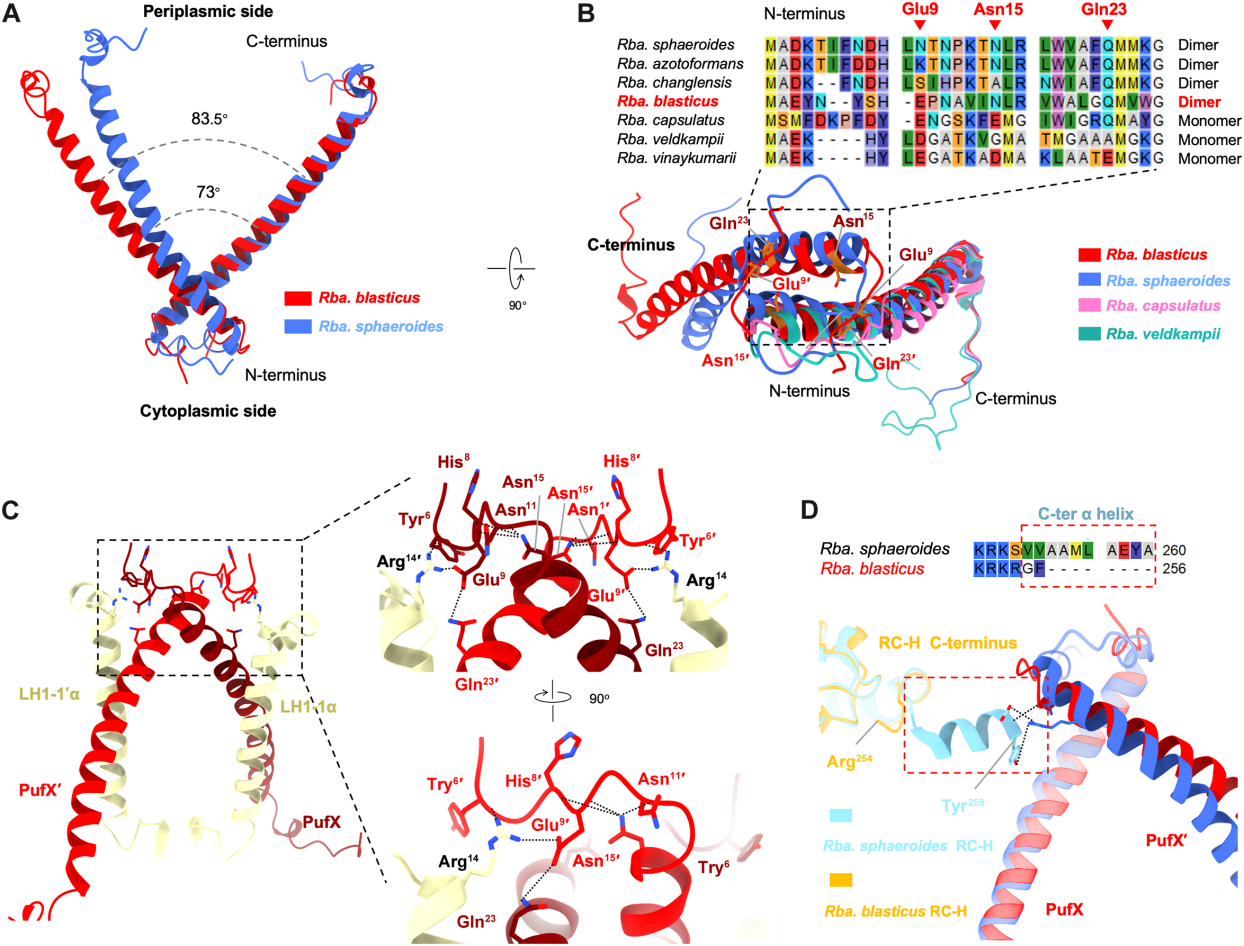
Structural basis for the dimerization of *Rba. blasticus* RC-LH1. (**A**) Structural alignment of the PufX dimers from *Rba. blasticus* and *Rba. sphaeroides*. (**B**) Structural and sequence alignments of the N-terminal regions of PufX from *Rba. blasticus*, *Rba. sphaeroides*, *Rba. capsulatus*, and *Rba. veldkampii*. (**C**) Interactions at the dimerization interface of *Rba. blasticus* RC-LH1 dimer. PufX is colored in dark red, PufX′ is colored in red, and LH1-α and LH1-α′ are colored in wheat. Hydrogen bonds and salt bridges are shown using dashed lines. (**D**) Comparison of the interaction interface between RC-H and PufX of the *Rba. blasticus* and *Rba. sphaeroides* RC-LH1 dimers.

Our cryo-EM structure shows that the dimerization interface of the RC-LH1 supercomplex in *Rba. blasticus* involves two PufX polypeptides (Fig. 1B). These PufX polypeptides are positioned at an angle of approximately 45° from the RC toward the peripheral side of LH1 and are slightly bent within the membrane layer. The overall structure of PufX in *Rba. blasticus* resembles that of PufX in *Rba. veldkampii*, *Rba. sphaeroides*, and *Rba. capsulatus* (Fig. 2B and fig. S6). The crossing angle for the PufX dimer in *Rba. blasticus* is approximately 84°, which is greater than that of the *Rba. sphaeroides* PufX dimer (73°) (Fig. 2A). This corresponds to the more flattened conformation of the *Rba. blasticus* RC-LH1 dimer (Fig. 1B and figs. S2B and S9). More tilted RC-LH1 dimers exist in the vesicular intracytoplasmic membranes (ICMs) of *Rba. sphaeroides*, whereas relatively flattened conformation of the RC-LH1 dimers were observed in both *Rba. blasticus* that has lamellar ICMs (fig. S1) and *Rca. bogoriensis* that form vesicular ICMs (*22*, *23*). These results suggest that the curved dimerization of RC-LH1 is not a prerequisite for the formation of vesicular ICMs.

The N-terminal region of PufX has been proposed to be related to RC-LH1 dimerization in *Rba. sphaeroides* (*4*, *7*, *20*, *25*). The structure of the *Rba. blasticus* PufX N-terminal region (Asn^5^-Val^13^) were well resolved (fig. S8). The PufX N-terminal regions of *Rba. blasticus* and *Rba. sphaeroides* extend toward the other ones in the PufX dimers, distinct from those of *Rba. capsulatus* and *Rba. veldkampii* (Fig. 2B). It appears to have a limited number of polar interactions with the neighboring RC-LH1 monomer within *Rba. blasticus* RC-LH1 dimer. Specifically, the side chains of PufX Glu9 and LH1-1α Arg^14^ of the other monomer form a salt bridge, and the side chain of Arg^14^ and the backbone of Tyr^6^ of PufX form a hydrogen bond (Fig. 2C). In *Rba. sphaeroides*, extensive hydrogen bonds are formed between the N-terminal region of PufX and the adjacent RC-LH1 monomer within the dimer (*4*, *7*). In contrast, the RC-H of *Rba. blasticus* lacks the C-terminal α helix, resulting in the absence of hydrogen bonding interactions, similar to that seen between PufX and the C-terminal α-helix of RC-H in *Rba. sphaeroides* (Fig. 2D and fig. S5C). In addition to the interactions of the N-terminal region of PufX with LH1-1α of the adjacent monomer, direct hydrogen bonds were found at the interface of two PufX polypeptides (between Glu^9^ and Gln^23^) to facilitate the PufX dimerization (Fig. 2C), whereas no detectable polar interactions were identified at the same interface in the *Rba. sphaeroides* RC-LH1 dimer (*4*, *7*). In addition, the amino groups of Asn^15^ side chains form hydrogen bonds with the backbone oxygen atoms of His^8^, Glu^9^, and Asn^11^ within the same subunit (Fig. 2C). Therefore, it is postulated that Glu^9^, Asn^15^, and Gln^23^ of PufX are important for maintaining the dimerization of *Rba. blasticus* PufX and RC-LH1.

Within the *Rhodobacter* species, *Rba. sphaeroides*, *Rba. blasticus*, *Rba. azotoformans*, and *Rba. changlensis* produce RC-LH1 dimers and monomers, whereas *Rba. capsulatus*, *Rba. veldkampii*, and *Rba. vinaykumarii* generate exclusively RC-LH1 monomers (*3*–*9*, *12*, *24*). Sequence alignment reveals a high sequence similarity among the N-terminal regions of PufX from *Rba. azotoformans*, *Rba. changlensis*, and *Rba. sphaeroides* (Fig. 2B). In contrast, the low sequence similarity between the N-terminal regions of PufX from *Rba. blasticus* and *Rba. sphaeroides* is consistent with the differences in the interactions at the dimerization interfaces. Intriguingly, although Glu^9^ and Gln^23^ are conserved in the PufX polypeptides from both *Rba. blasticus* and *Rba. capsulatus*, the PufX polypeptide of *Rba. capsulatus* fails to dimerize (*8*). The discrepancy between the PufX subunits from *Rba. blasticus* and *Rba. capsulatus* is presumably due to the mutation of Asn^15^ in *Rba. blasticus* (Asn^20^ in *Rba. sphaeroides*, Glu^17^ in *Rba. capsulatus*, and Gly^14^ in *Rba. veldkampii*) (Fig. 2C). This mutation may compromise the hydrogen bonding and affect the positioning of Glu^9^, likely leading to the failure of dimerization.

**Fig. 3.**
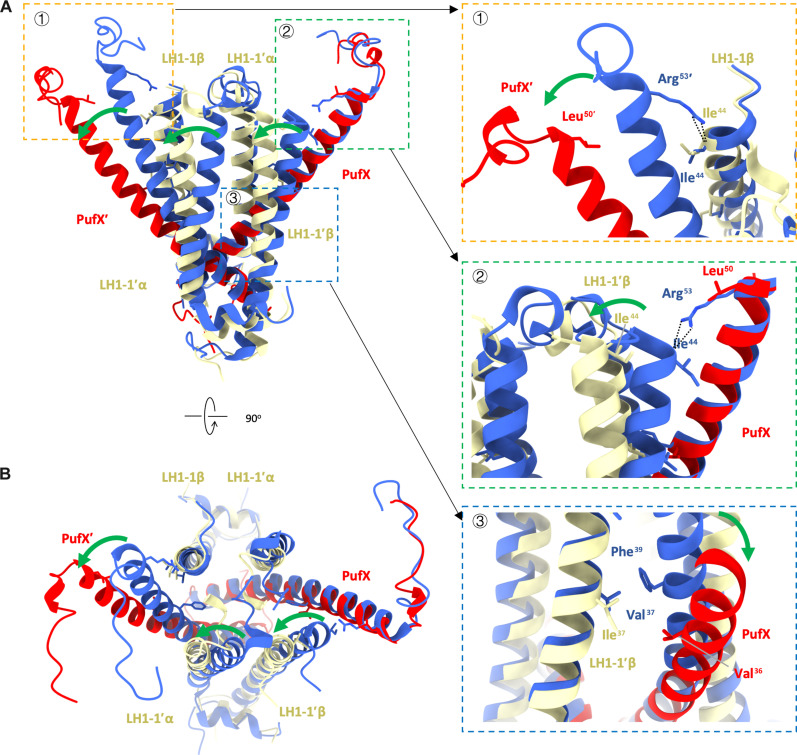
Structural basis for the different crossing angles between *Rba. blasticus* and *Rba. sphaeroides* RC-LH1 dimers. (**A**) Side view of the structural alignment, with close-up views of the interactions related to the differences in crossing angles shown on the right. (**B**) View of the structural alignment from the periplasmic side. Mutations in key residues, such as Leu^50^ and Ile^37^ in the *Rba. blasticus* RC-LH1 dimer, resulted in differences in the crossing angles. The *Rba. sphaeroides* RC-LH1 dimer is colored in blue. The PufX and PufX′ of the *Rba. blasticus* RC-LH1 dimer are colored in red, while the LH1-αβ are colored in wheat. Green arrows indicate the direction of α helix deflection, and dashed lines show the hydrogen bonds.

A notable difference between the PufX dimer arrangements of *Rba. blasticus* and *Rba. sphaeroides* is their crossing angles (Fig. 2A). The alternation in the crossing angle of the PufX dimer is likely associated with the structural changes in the C-terminal and transmembrane regions of PufX. These regions are separated by LH1-1 from each monomer and do not have any close contacts with each other (Fig. 3, A and B). Previous studies have suggested that *Rba. sphaeroides* LH1-1 from the neighboring RC-LH1 monomer plays a crucial role in determining the positions of the transmembrane and C-terminal regions of PufX (*4*, *7*). Sequence alignment shows that PufX of *Rba. blasticus* has mutations at Val^36^ and Leu^50^ compared to *Rba. sphaeroides* PufX (fig. S6A). These mutations break hydrogen bonds between the Arg^53^ side chain in the C-terminal region of *Rba. sphaeroides* PufX and the Ile^44^ backbone of LH1-1′β, as well as CH-π interactions between Phe^39^ in the transmembrane region of PufX and the Val^37^ methyl group of LH1-1′β (*4*). These changes could potentially result in a greater separation between PufX and the neighboring LH1-1αβ (Fig. 3A), ultimately leading to variations in the crossing angles of RC-LH1 dimers between *Rba. blasticus* and *Rba. sphaeroides*. Furthermore, superimposition of RC-LH1 monomers from *Rba. blasticus* and *Rba. sphaeroides* indicates that their PufX subunits have similar positions with respect to other subunits within the monomers (fig. S6). Overall, our results indicate that the difference in the crossing angle of the PufX dimers is primarily influenced by changes in the interactions between PufX and the neighboring RC-LH1 monomer, rather than the interactions of PufX with RC-LH1 subunits within the same monomer. Advanced knowledge of the interactions involved in RC-LH1 dimerization may inform the future engineering of RC-LH1 to create photosynthetic complexes with tailored architectures, stability, and functionality.

### Intra- and intersubunit interactions within LH1

A typical LH1 subunit comprises an LH1-αβ heterodimer that sandwiches two bacteriochlorophylls (BChls) *a* and two spheroidenes (SPOs) (fig. S11). The key interactions maintaining an LH1 αβ heterodimer are predominantly located in the N- and C-terminal regions of LH1 αβ polypeptides located on the cytoplasmic and periplasmic sides, respectively. In the N-terminal region, the side chains of Asp^14^ and His^21^ in LH1-β form hydrogen bonds or salt bridges with the side chains of Lys^6^, Gln^9^, and Trp^8^ in the LH1-α N-terminal short helix (fig. S11A). In the C-terminal region, the side chain of Arg^46^ and the oxygen atom of Pro^47^ in LH1-β form hydrogen bonds or salt bridges with the side chains of Tyr^44^ and Asp^40^ in the LH1-α C-terminal short helix. In the transmembrane region, hydrogen bonds were also determined between the side chain of LH1-α Tyr^24^ and the side chain of LH1-β Gln^20^. Furthermore, pigment molecules within the LH1 subunit also contribute to the intrasubunit interactions of the LH1-αβ heterodimer. His^32^ and Trp^43^ of LH1-α, as well as His^39^ and Trp^48^ of LH1-β, form hydrogen bonds with BChls within the LH1 subunit (fig. S11A).

The intersubunit interactions between LH1 heterodimers are also formed mostly at the LH1-α N- and C-terminal helices. LH1-α and LH1-β form hydrogen bonds with their neighboring LH1-β_n+1_, LH1-β_n−1_, and LH1-α_n−1_. The interaction between LH1-α_n_ and LH1-β_n+1_ relies mainly on the only observed hydrogen bond formed between the oxygen on the main chain of Met^1^ in the LH1-α_n_ N-terminal short helix and the side chain of Ser^26^ on LH1-β_n+1_ (fig. S11B). On the other hand, extensive hydrogen bonds were observed among the N-terminal regions of LH1-α_n_, LH1-β_n−1_ and LH1-α_n−1_. The side chains of Arg^53^ and Tyr^51^ in LH1-α_n_ are hydrogen bonded to the oxygen on the main chain of Pro^47^ and Leu^49^ in LH1-β_n−1_, respectively (fig. S11B). In addition, the side chain groups of Asp^45^ and Arg^53^ in LH1-α_n_ form hydrogen bonds or salt bridges with Lys^38^ and Tyr^41^ of LH1-α_n−1_, respectively. The LH1 interactions within the dimeric and monomeric structures resemble those determined in *Rba. veldkampii*, *Rba. sphaeroides*, and *Rba. capsulatus* RC-LH1complexes (*3*, *4*, *8*).

### Cofactors in RC-LH1

**Fig. 4.**
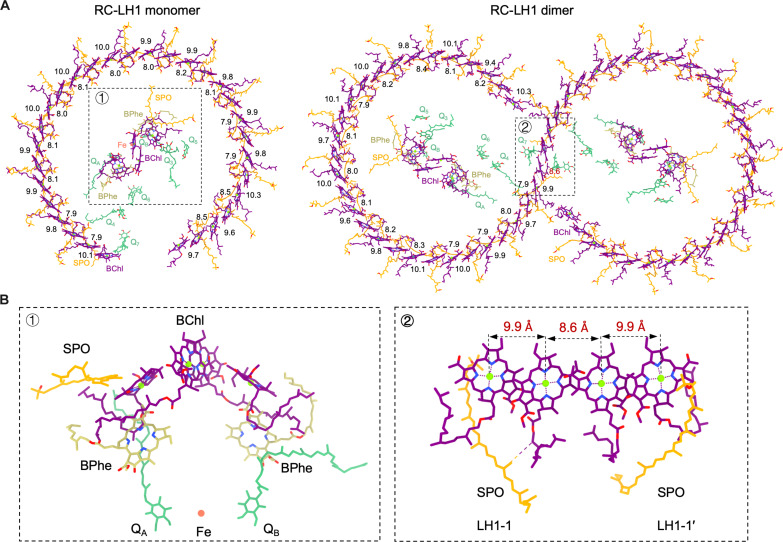
Cofactors arrangement of the RC-LH1 monomer and dimer from *Rba. blasticus*. (**A**) Arrangement of cofactors within the RC-LH1 monomer and dimer from the periplasmic view. The Mg-Mg distances in Ångstroms for intra- and intersubunit BChls are indicated. BChls are depicted in purple, SPOs in orange, BPhes in yellow, and UQ-10s in lawn green. (**B**) Closer views of the arrangement of cofactors associated with the RC (left) and dimerization interface of the RC-LH1 dimer (right).

In the RC-LH1 monomer of *Rba. blasticus*, a total of 34 BChls *a*, 29 SPOs, two bacteriopheophytins (BPhes), 7 ubiquinone-10 (UQ-10) molecules, and one nonheme Fe were identified (Fig. 4A). The RC contains a BChl *a* pair as the primary electron donors, two BChl *a* monomers, one SPO, two BPhes, two UQ-10 molecules (Q_A_ and Q_B_), and an Fe^2+^ ion. The LH1 ring comprises 30 BChls *a*. The intra-subunit Mg-Mg distances range from 9.6 to 10.3 Å, whereas the Mg-Mg distances of BChls associated with neighboring LH1 subunits are slightly shorter, ranging from 7.9 to 8.5 Å (Fig. 4A).

**Fig. 5.**
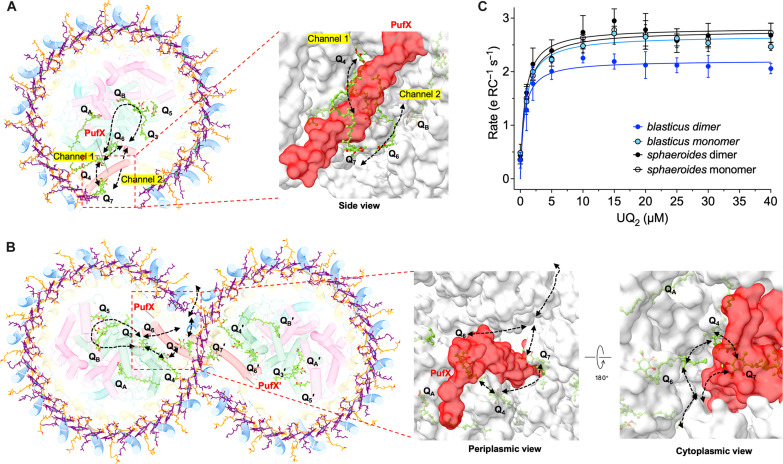
Proposed pathways for quinone/quinol exchange in the RC-LH1 supercomplexes. (**A**) Proposed pathways for quinone/quinol exchange in the RC-LH1 monomer based on the structural model. The surface view of the quinone/quinol channels for the entry and exit of the RC-LH1 monomer is depicted at the bottom. The arrows indicate the diffusion pathways for quinone/quinol. (**B**) Proposed pathways for quinone/quinol exchange in the RC-LH1 dimer. The surface view of the quinone/quinol channels for the entry and exit of the RC-LH1 dimer is presented at the bottom. The arrows indicate the pathways for quinone/quinol diffusion. BChls are colored in purple, SPOs in orange, and UQ-10s in lawn green. (**C**) Kinetics of cytochrome *c*_2_ oxidation of RC-LH1 dimers and monomers from *Rba. blasticus* upon illumination in the presence of various concentrations of UQ-2, in comparison with that of *Rba. sphaeroides* RC-LH1 counterparts (see also fig. S17). Data were obtained on the basis of three independent biological replicates.

Each LH1 typically accommodates two SPOs (SPO-α and SPO-β), except for LH1-1 and LH1-15, which lack one SPO. In LH1-1, the absence of SPO-β is attributed to the steric hindrance caused by PufX. The presence of PufX influences the conformation of the phytol tail of the adjacent BChl, leading to the occupation of the SPO-β binding site, thereby affecting SPO-β attachment (fig. S12). The exact number of SPOs coordinated within LH1-15 could not be well determined based on the limited local density (figs. S2 and S12B). Hence, only SPO-β was modeled for LH1-15. The arrangement of SPOs is similar to that of *Rba. sphaeroides* and *Rba. capsulatus* but different from *Rba. veldkampii*, which only contains one SPO within each LH1 heterodimer (*3*–*9*). The extensive interactions of SPOs with LH1 subunits and bound BChls ensure the stabilization of carotenoids within LH1 and the transfer of excitation energy between carotenoids and BChls in the LH1 ring (Figs. 4 and 5A).

The dimeric RC-LH1 of *Rba. blasticus* contains double the number of cofactors as the monomeric RC-LH1, and the locations of these pigment molecules are similar. The intrasubunit Mg-Mg distances of the dimeric RC-LH1 range from 9.4 to 10.3 Å, while the Mg-Mg distances of BChls associated with neighboring LH1 subunits range from 7.9 to 8.4 Å (Fig. 4A). LH1-1 and LH1-1′ from neighboring monomers are closely associated, characterized by BChls with Mg-Mg distances of approximately 8.6 Å (Fig. 4B).

The special pair of BChls in the RC is aligned at the same level as the LH1 ring in the transmembrane region and has relatively equal distances to the closest LH1 BChls (35.5 to 47.3 Å for monomer, 35.7 to 47.1 Å for dimer), which are comparable to those of the *Rba. sphaeroides* RC-LH1 monomer and dimer (fig. S13) (*4*, *26*). This may provide the structural basis for efficient excitation energy transfer (EET) from LH1 subunits to the RC. Consistently, transient absorption (TA) spectra showed that lifetime density kinetic traces (LDKTs), which are dominated by LH1 → RC EET, have peak lifetimes (τ_EET_) of 42 and 41 ps for the *Rba. blasticus* RC-LH1 monomer and dimer, respectively, which are on similar timescales as the τ_EET_ of the *Rba. sphaeroides* RC-LH1 monomer and dimer (45 and 42 ps, respectively) (fig. S14). These are in good agreement with the values reported recently for *Rba. sphaeroides* RC-LH1 (*26*), as well as other RC-LH1 structures (*11*, *27*). We have recently highlighted that the distribution of EET lifetimes observed in LDKT is reflective of the degree of disorder within the RC-LH1 structures, particularly the orientation of the RC, during our room temperature TA experiments. The distributions of the lifetimes observed for all four RC-LH1 complexes are remarkably similar, indicating that the RC is well constrained within the LH1 ring in *Rba. blasticus* despite the absence of PufY.

### Possible pathways for quinone/quinol exchange in *Rba. blasticus* RC-LH1

Within the RC-LH1 monomer of *Rba. blasticus*, five extra UQ-10 molecules (Q_3_ to Q_7_) were identified, in addition to Q_A_ and Q_B_ in the RC (Fig. 5A). Among them, Q_3_ and Q_5_ are potential UQ-10 molecules located in the gap between the LH1 ring and the RC. Q_3_ is positioned near the periplasmic surface with its head group protruding toward the isoprenoid tail of Q_B_ (Fig. 5A and fig. S15). Consistently, UQ-10 molecules have been identified in the similar positions within the RC-LH1 complexes of *Rba. sphaeroides*, *Rba. capsulatus*, and *Rba. veldkampii* (*3*, *4*, *8*). Q_5_ is situated close to Q_3_ and faces the periplasm in a direction opposite to that of Q_3_ (Fig. 5A and fig. S15), sharing a similar position as Q_6_ in *Rba. veldkampii* and Q_P_/Q_D_ in *Rba. capsulatus* (*3*, *8*, *9*). Q_4_ is sandwiched among RC-L, PufX, and LH1-1. A UQ-10 molecule was also identified in a similar location within the *Rba. veldkampii* RC-LH1 monomer (namely, Q_5_) (*3*). However, Q_4_ is situated closer to PufX than Q_5_ in *Rba. veldkampii* RC-LH1, with its head group near the periplasmic surface, and its isoprenoid tail is not wedged between the first and second LH1 αβ heterodimers (Fig. 5A and figs. S15 and S16). Furthermore, two previously unidentified UQ-10 molecules (assigned as Q_6_ and Q_7_) were found in both the *Rba. blasticus* RC-LH1 monomers and dimers (Fig. 5, A and B). Q_6_ is located in the cavity among RC-L, RC-H, PufX, and LH1-15, while Q_7_ is located at the peripheral side of PufX within the same RC-LH1 monomer. Both the head groups of Q_6_ and Q_7_ are oriented toward the cytoplasmic side (Fig. 5, A and B, and fig. S15). Note that definitively distinguishing Q_4_ and Q_7_ from detergent molecules based on the current local densities is difficult, which merits further investigation.

On the basis of the structural analysis, the possible quinone channels in the *blasticus* RC-LH1 complex were proposed. PufX mediates the formation of a large opening between the LH1-1 and LH1-15 subunits of the *Rba. blasticus* RC-LH1 monomer. It also splits the opening into two channels (channel 1 and channel 2) (Fig. 5A and fig. S15). Both channels appear to offer sufficient space for quinone/quinol exchange, as Q_4_, Q_6_, and Q_7_ were identified around the periphery of these two channels near PufX. In the RC-LH1 complexes that contain only one SPO per LH1 subunit, such as *Rba. veldkampii* RC-LH1 monomer, the lack of one SPO molecule results in a small pore between two adjacent LH1 subunits, which is presumably a putative channel for quinone diffusion (fig. S16). In contrast, this channel is blocked by the presence of two SPOs between LH1 subunits in the *Rba. blasticus* RC-LH1 monomer. Consistently, the isoprenoid tail of Q_4_ is not inserted between LH1 αβ-heterodimers, differing from Q_5_ in *Rba. veldkampii* RC-LH1 (Fig. 5 and fig. S16). Likewise, in the RC-LH1 complexes of *Rba. capsulatus* and *Rba. sphaeroides*, which contain two SPOs per LH1 subunit (*4*–*9*), no UQ-10 molecule was identified in the same location as *Rba. blasticus* Q_4_.

Although both the RC-LH1 dimers of *Rba. blasticus* and *Rba. sphaeroides* have S-shaped LH1 rings, the *Rba. blasticus* RC-LH1 dimer has a more closed LH1 ring than the *Rba. sphaeroides* counterpart owing to the incorporation of the additional LH1-15 αβ heterodimers (Fig. 5B and fig. S9). Consequently, the LH1 opening of the *Rba. blasticus* RC-LH1 dimer is significantly narrower than those of the *Rba. sphaeroides* RC-LH1 dimer (fig. S9). As mentioned earlier, the poor local density did not allow us to ascertain whether LH1-15 is missing SPO-α. Nevertheless, we speculated that LH1-15 probably lacks SPO-α, and this absence may facilitate quinone trafficking. To evaluate the effects of the LH1 opening on quinone/quinol diffusion, we conducted cytochrome *c* oxidation assays on the RC-LH1 dimers and monomers from *Rba. blasticus* and *Rba. sphaeroides*. Our results reveal that the maximum quinone/quinol exchange rate of the *Rba. blasticus* RC-LH1 monomer [2.7 ± 0.2 e^−^ RC^−1^ s^−1^, *K*_m_ (Michaelis constant) = 0.72 ± 0.3 μM, *N* = 3] was comparable to that of the *Rba. sphaeroides* monomer (2.8 ± 0.3 e^−^ RC^−1^ s^−1^, *K*_m_ = 0.78 ± 0.3 μM, *N* = 3), indicating that the reduced LH1 opening of the *Rba. blasticus* RC-LH1 monomer, due to the presence of LH1-15, could still offer sufficient channel space for quinone diffusion (Fig. 5C and fig. S17). In contrast, the maximum quinone/quinol exchange rate of the *Rba. blasticus* RC-LH1 dimer (2.2 ± 0.1 e^−^ RC^−1^ s^−1^, *K*_m_ = 0.59 ± 0.2 μM, *N* = 3) was 21% slower than that of *Rba. sphaeroides* RC-LH1 dimer (2.8 ± 0.2 e^−^ RC^−1^ s^−1^, *K*_m_ = 0.70 ± 0.3 μM, *N* = 3). This suggests that the narrower LH1 opening in the *Rba. blasticus* RC-LH1 dimer, resulting from the presence of LH1-15 and the LH1 barrier of adjacent monomer, notably hinders quinone migration. However, with the current analytical method, we cannot exclude the possibility that the use of UQ-2 and detergents in the RC-LH1 sample solution may potentially enable quinone exchange through channels other than the LH1 opening, which awaits further investigation.

### Insights into the architectural diversity and modularity of *Rba.* RC-LH1 complexes

Photosynthetic complexes use diverse strategies to fulfill the demands of light absorption and quinone/quinol transport (*10*, *28*). Structural analysis of RC-LH1 from *Rba. blasticus* and other reported RC-LH1 complexes offer the opportunities for assessing the structural variations of RC-LH1 complexes of the *Rba.* genus. Some *Rba.* RC-LH1 complexes contain two carotenoids per LH1 subunit. While the incorporation of additional carotenoid enhances light capture and photoprotection, it also provides spatial obstruction that impedes quinone/quinol exchange across the LH1 ring through the pores formed between adjacent LH1 subunits. The evolution of PufX in many strains of the *Rba.* genus, such as *Rba. sphaeroides* and *Rba. capsulatus*, may address this issue by forming a wider opening in the LH1 ring to facilitate quinone/quinol diffusion (fig. S18). In contrast, the RC-LH1 monomer of *Rba. veldkampii* contains PufX and only one carotenoid per LH1 subunit, presumably representing a transitional state of the *Rba.* genus (fig. S18). On the other hand, the PufX-mediated LH1 opening leads to a decrease in the number of LH1 subunits, which may result in a reduced capacity for light harvesting. The dimerization of RC-LH1, such as in *Rba. blasticus*, may enhance the quantum efficiency of energy trapping by concentrating pigments within the local membrane area and ensuring spillover of excitons to adjacent RC-LH1 if one RC has undergone photochemical charge separation (*12*). However, a drawback could be the reduction in the rate of quinone turnover ascribed to the narrowed opening in the S-shaped LH1 ring. Compared to that of *Rba. blasticus*, the *Rba. sphaeroides* RC-LH1 dimer contains an additional subunit, PufY, which further enlarges the LH1 opening. These structural properties lead to the absence of the last two LH1 subunits, thereby expanding the channels for quinone/quinol diffusion. Furthermore, to compensate for the reduction in the number of LH1 subunits which may lead to reduced light harvesting, the *Rba. sphaeroides* RC-LH1 dimer adopts a more bent architecture (fig. S9), which induces the curvature of photosynthetic membranes for enhanced surface area and local density of photosynthetic complexes per membrane area, ultimately improving the light-harvesting capacity of the photosynthetic machinery. All these structural features underscore the structural variability and modularity of native photosynthetic RC-LH1 supercomplexes and the photosynthetic apparatus in response to environmental changes. Gaining deeper insight into the structure and function of RC-LH1 will facilitate the engineering of proteins and photosynthetic organisms to create RC-LH1 with specific architecture and stability. This knowledge will inform the rational design and bioengineering of photosynthetic complexes for diverse applications including solar energy conversion, biofuel production, and biomimetic light-harvesting systems.

## MATERIALS AND METHODS

### Growth condition of *Rba. blasticus*

WT *Rba. blasticus* LMG 4305 (accession number: LMG 4305) was obtained from Belgian Coordinated Collections of Microorganisms. The *Rba. blasticus* cells were grown phototrophically under anoxic conditions in liquid magnesium–calcium, peptone, yeast extract (MPYE) medium supplemented with vitamins (0.08 M nicotinic acid, 0.01 M thiamine, 7.3 mM 4-aminobenzoic acid, and 0.4 mM d-biotin) at 30°C in sealed glass bottles under a light intensity of 25 μmol photons s^−1^ m^−2^ (Bellight 70-W halogen bulbs).

### Thin-section transmission electron microscopy

Cells were pelleted by centrifugation (6000*g*, 10 min) and processed for thin section using a Pelco BioWave Pro laboratory microwave system. The cells were first fixed with 0.1 M sodium cacodylate buffer (pH 7.2) supplemented with 2% glutaraldehyde using two steps of 100 W for 1 min each (P1). Samples were then embedded in 4% agarose, followed by staining with 2% osmium tetroxide and 3% potassium ferrocyanide using three steps of 100 W for 20 s each (P2). The reduced osmium stain was then set in a 1% thiocarbohydrazide solution for 10 min. The second osmium stain was applied using P2 with 2% osmium tetroxide. The sample was made electron dense by incubation with 2% uranyl acetate at 4°C overnight. Dehydration was operated with a series of increasing alcohol concentrations (30 to 100%) before cells were embedded in medium resin. Thin sections of 70 nm were cut with a diamond knife, followed by a post-stain with 3% lead citrate. Images were recorded on an FEI 120-kV Tecnai G2 Spirit BioTWIN transmission electron microscope (FEI, USA) equipped with a Gatan Rio 16 camera and the DigitalMicrograph software (Gatan, USA).

### Purification of RC-LH1 complexes

Cells were harvested by centrifugation at 5000*g* for 10 min at 4°C, washed three times with tris-HCl (pH 8.0), and resuspended in 20 mM Hepes (pH 8.0). Cells were disrupted by passage through a French press three times at 16,000 psi. Cell debris was removed by centrifugation at 20,000*g* for 30 min. Membranes were collected by centrifuging the resulting supernatant at 125,000*g* for 90 min and were solubilized by addition of β-DDM (n-dodecyl β-d-maltoside) (Melford, catalog no. D12000) to a final concentration of 3% (w/v) for 30 to 60 min in the dark at 4°C with gentle stirring. Unsolubilized protein material was removed by centrifugation at 21,000*g* for 30 min. Then, the supernatant was applied onto the 10 to 25% (w/v) continuous sucrose gradients made with working buffer containing 0.01% (w/v) β-DDM. Gradients were centrifuged at 230,000*g* for 18 hours. The RC-LH1 complexes in the sucrose gradient solution were collected, and the purity of RC-LH1 complexes was characterized by SDS–polyacrylamide gel electrophoresis and absorption spectra (fig. S1).

### Absorption spectra

Purified RC-LH1 complexes were collected from sucrose gradients, and absorbance was measured from 300 to 900 nm at 1-nm intervals using a Libra S22 spectrophotometer (Biochrom, UK).

### TA spectra

TA spectroscopy was conducted using a Harpia-TA spectrometer (Light Conversion). The PHAROS-SP 10-W laser system (Light Conversion) produced the probe and pump, operating at 1028 nm with a frequency of 10 kHz and a full width at half maximum of approximately 170 fs. The pump beam is tuned to the desired wavelength using an optical parametric amplifier (OPA) (Orpheus, Light Conversion) and with a beam diameter of ca. 600 μm (1/e^2^ diameter) at the sample. The pump beam is chopped, resulting in an effective pump rate of 5 kHz. The white light probe is generated by focusing the 1028-nm beam onto a sapphire crystal and is focused to ca. 400-μm beam at the sample. The pump polarization was altered to ensure that the pump and probe beam interact with the sample at the magic angle of 54.7° to eliminate the effect of anisotropy and rotational diffusion on the spectra. Spectra were recorded using an n-channel metal oxide semiconductor (NMOS) detector (S3901, Hamamatsu) after dispersion by a spectrograph (Kymera 193i, Andor), allowing probing within the 530 to 950 nm in range. A pump power of 50 μW (effective pumping rate of 5 kHz) was used for all samples which reduced exciton-exciton annihilation effects while maintaining a good signal to noise ratio required for data analysis.

The experiments were performed using a 2-mm pathlength quartz cuvette; the solution, left undisturbed during for the duration of each experiment (1 hour 20 min) and the signal remained stable. Samples were diluted to OD of ~0.1 in IMAC buffer containing 50 mM sodium ascorbate, which served as a sacrificial electron donor. The pump wavelength was chosen to match the absorption maximum of the ^LH1^BChl (Q_y_) band observed in the steady-state ultraviolet (UV)/visible spectrum for each RC-LH1 supercomplex. Before each experiment, the sample underwent 60 s of pump beam irradiation to guarantee that the RC Q_A_ was photochemically reduced, enabling the investigation of Q_A_ inactivation in the charge transfer relaxation process within the RC.

The data were initially processed using CarpetView (Light Conversion) to account for chirp correction (performed using the response from a silicon wafer). Because of the experimental setup, scattered pump signals were detected. To account for this, a 15-nm range on either side of the pump wavelength was excluded before subsequent analysis. Lifetime density analysis (LDA) was performed using OPTIMUS (*29*). Only the 750- to 950-nm spectral region, which contains the dominating TA features, was included in the LDA fit; this considerably reduced computational resources required and prevented data smoothing owing to the introduction of low signal:noise data (as a result of the weak TA features observed <750 nm). In all cases, data were fitted by using three Gaussian coherent artifact signals. Inspection of lifetime density maps resulting from LDA is complicated owing to the difficulty in accurately representing the pre-exponential factor magnitude with contour/color maps. We reduced the three-dimensional (3D) lifetime density map to 2D kinetic traces by integrating the modulus of the pre-exponential factor between 750 and 950 nm for each lifetime. This generated what we term a LDKT (fig. S14). By calculating the wavelength-dependent average pre-exponential factor of lifetimes associated with the dominant band in the LDKT with a peak at ~40 ps, we plot the spectral change associated with this kinetic process in two dimensions, which we term “lifetime averaged difference spectra”.

### Cytochrome *c* oxidation assays

Assays were conducted using RC-LH1 complexes with a concentration of 30 nM, incorporating 30 μM reduced horse-heart cytochrome *c*_2_ (Merck, catalog no. C2506, UK) and varying concentrations of UQ-2 (Merck, catalog no. C8081, UK) ranging from 0 to 50 μM in Hepes buffer (0.01% β-DDM, pH 8.0). Three, 1 ml of sample replicates at each UQ-2 concentration was incubated at 4°C in the dark overnight to ensure complete dark adaptation before measurements. Measurement was carried using Cole-Parmer SP-800-UV spectrophotometer. To exclude the excitation light, sample and reference detector entrances are equipped with 550-nm short pass (Thorlabs, FES0550). Absorbance was monitored at 550 nm with 1-s interval time. Excitation light of 40 μW is provided by an 880-nm light-emitting diode (Thorlabs, M880F2) and delivered at 90° angle to the measurement beam. Absorbance was recorded for 1 min before a 5-min illumination period. Data were processed by fitting of the linear initial rate before the equilibrium phase, over 1 to 10 s (dependent on UQ-2 concentration) and averaging the rates of all three samples at each UQ-2 concentration. Rates were converted into catalytic efficiencies using the concentrations of RC-LH1 dimers and reduced cytochrome *c*_2_ calculated with their respective extinction coefficients and were fitted with the Michaelis-Menten model to determine apparent *K*_m_ and *K*_cat_ values using Origin Pro 2021b (OriginLab, USA).

### Cryo-EM data collection

A 3.0-μl aliquot of the RC-LH1 complex was applied onto the holey carbon grids (Quantifoil R1.2/1.3 Au, 200 mesh) that were glow discharged (20 mAmp, 40 s, Quorum GloQube). The grids were plunge-frozen into liquid ethane using an FEI Vitrobot Mark IV (Thermo Fisher Scientific) at 4°C and >90% humidity with 5- to 7-s blot time and 0 blot force. Data collection was conducted on a 300-kV KriosG4 Cryo-TEM (Thermo Fisher Scientific) equipped with a low-energy spread cold field emission gun (E-CFEG), a Thermo Scientific Selectris X Energy Filter, and a Falcon 4 Detector. The Thermo Scientific Smart EPU software was used to collect movies of the RC-LH1 complexes with a pixel size of 0.929 Å, 10-eV slit width, a total dose of 40 e/Å^2^, and defocus range of −0.8 to −2 μm using Aberration-free image shift. A total of 6618 movies for RC-LH1 monomers and 2281 movies for RC-LH1 dimers were collected for further data processing.

### Data processing

Data processing was performed using the cryoSPARC software (*30*). For the monomer dataset, after patch motion and contrast transfer function (CTF) correction were performed, the micrographs that yielded worse than 4.5-Å resolution were discarded. Of the remaining 6550 micrographs, 1,476,836 particles were picked using Blob picking algorithm of cryoSPARC. The particles were extracted, 4× binned, and subjected to three rounds of 2D classification. These runs yielded ~85,696 particles for analysis, which were subjected to Ab initio reconstruction with four classes. Three classes were selected and re-extracted as unbinned to perform another round of Ab initio reconstruction with three classes. Last, one “best-looking class” was selected to perform non-uniform refinement in cryoSPARC. The final number of particles was 45,399, and the resolution was 2.72 Å.Another round of non-uniform refinement was performed using “optimize per-particle defocus” option, which resulted with a 2.6-Å resolution final map. Local map resolutions were calculated by cryoSPARC.

For the dimer dataset, after patch motion and CTF correction were performed, a total of 524,356 particles were picked using Blob picking algorithm of cryoSPARC. Two rounds of 2D classification yielded 13,544 particles for further analysis. Ab initio reconstruction with two classes yielded one “good-looking” class with 9149 particles. The final particles were subjected to non-uniform refinement with C1 and C2 symmetry, which yielded 3.22 and 3 Å, respectively. C2 symmetry-imposed map was subjected to another non-uniform refinement with optimize per-particle defocus option. The final resolution resulted in 2.84 Å. Local map resolutions were calculated by cryoSPARC.

### Model building and refinement

The structure of RC-LH1 monomer from *Rba. sphaeroides* [Protein Data Bank (PDB) ID: 7VNY] was initially docked into the resolution cryo-EM map of the RC-LH1 monomer of *Rba. blasticus* using UCSF Chimera (*31*). The amino acid sequences were further mutated to its counterparts in *Rba. blasticus*. The model of RC-LH1 monomer was rebuilt manually basing on the cryo-EM density with COOT (*32*) and then real-space refined using Phenix 1.16.3549 (*33*). The atomic model of the RC-LH1 dimer was built using a similar method with the RC-LH1 monomer but using the RC-LH1 monomer as a reference starting model. Manually refined model was then refined using Phenix 1.16.3549. MolProbity 4 (*34*) was used to evaluate the geometries of the structures. Images were rendered with Chimera and ChimeraX (*35*).

### MD simulations

The initial model for the molecular dynamic simulation of RC-LH1 dimer was prepared as follows. First, the N- and C terminus of the protein chain were capped using acetyl (ACE) and N-methyl (NME) groups, respectively. Then, the RC-LH1 dimer was embedded in a pre-equilibrated lipid bilayer consisting of a single-component 1-palmitoyl-2-oleoyl-*sn*-glycero-3-phosphocholine (POPC) based on the predicted position from the positioning of proteins in membrane server (*36*). Moreover, the system was solvated in a TIP3P bilayer water shell and neutralized by adding 14 chloride ions. Last, 0.15 mM NaCl was added to simulate its physiological salt concentration. The generated system was composed of 68 protein chains, 68 BChls, 4 BPhes, 4 cardiolipin (CDL), 52 SPO, 14 UQ-10, and 849 POPC molecules, as well as 2 Fe^2+^, 294 Na^+^, 308 Cl^−^ ions, and 118,665 water molecules. The total number of atoms was 564,945, and the system size was 307.3 × 176.5 × 115.3 Å.

The AMBER ff19SB force field parameter set (*37*) was selected for standard amino acids residues. Water molecules were modeled using the TIP3P model (*38*). The force field parameters of POPC were adopted from Lipid17 [unfortunately only Lipid14 (*39*) has been published]. The histidine residues in the system were singly protonated on Nε, except for those ligated to BCL or Fe^2+^ via Nε (and thus protonated on Nδ). The parameters for BChl and BPhe were taken from previous studies (*40*). For clusters consisting of Fe^2+^ with its coordination residues (four histidines and one glutamate), the force field parameters were obtained using the procedure developed previously (*41*). For the cofactors CDL, SPO, and UQ-10, the generalized amber force field parameter set (*42*) was adopted, and the atomic charges were determined by fitting the electrostatic potential around these molecules using the restrained electrostatic potential (RESP) model (*43*).

Energy minimization of the whole system was performed with a three-step procedure: (i) The positions of all phospholipids, water molecules, all additive residues and all hydrogen atoms were minimized with 10,000 steps with the steepest descent algorithm by freezing the rest of the system. (ii) Ten thousand steps of energy minimization were performed for the whole system with restraints (100 kcal mol^−1^ Å^−2^) on the protein backbone and heavy atoms of cofactors. (iii) Ten thousand steps of energy minimization were performed without any restraints. The whole system was then slowly heated to 300 K within 120 ps under the restraint (10 kcal mol^−1^ Å^−2^) applied to the protein backbone atoms, the heavy atoms of cofactors, and phospholipid headgroups. After these optimization and heating procedures, a 40-ns equilibrium simulation was performed under the nPT ensemble at 300 K to balance the dimensions and density of the system. Last, a 500-ns production MD simulation was performed using the graphics processing unit (GPU) implementation of PMEMD from the AMBER16 software package (*44*). During the equilibrium and production MD simulations, a constraint (100 kcal mol^−1^ Å^−2^) is applied to the protein backbone to maintain its conformation. Atomic coordinates of all atoms were recorded every 1 ps. The temperature was controlled using the Langevin thermostat (*45*), while pressure was controlled using the anisotropic Berendsen barostat (*46*). Bond lengths involving hydrogen atoms were constrained using the SHAKE algorithm (*47*). Van der Waals interactions were treated using a nonbonding cutoff of 10 Å, and electrostatic interactions were calculated with the particle mesh Ewald method (*48*).
