# Supplementary material for: Architectures of photosynthetic RC-LH1 supercomplexes from Rhodobacter blasticus
Source: Sci Adv. 2024 Oct 9;10(41):eadp6678. doi: 10.1126/sciadv.adp6678 (PMC11463270; doi:10.1126/sciadv.adp6678)
Supplement: Supplementary file 1 — Figs. S1 to S18 Table S1 [file sciadv.adp6678_sm.pdf]

Science Advances
AAAS

Supplementary Materials for

Architectures of photosynthetic RC-LH1 supercomplexes from *Rhodobacter blasticus*

Peng Wang *et al.*

Corresponding author: Jun Gao, gaojun@mail.hzau.edu.cn; Yu-Zhong Zhang, zhangyz@sdu.edu.cn; Lu-Ning Liu, luning.liu@liverpool.ac.uk

Sci. Adv. **10**, eadp6678 (2024)
DOI: 10.1126/sciadv.adp6678

This PDF file includes:

Figs. S1 to S18
Table S1

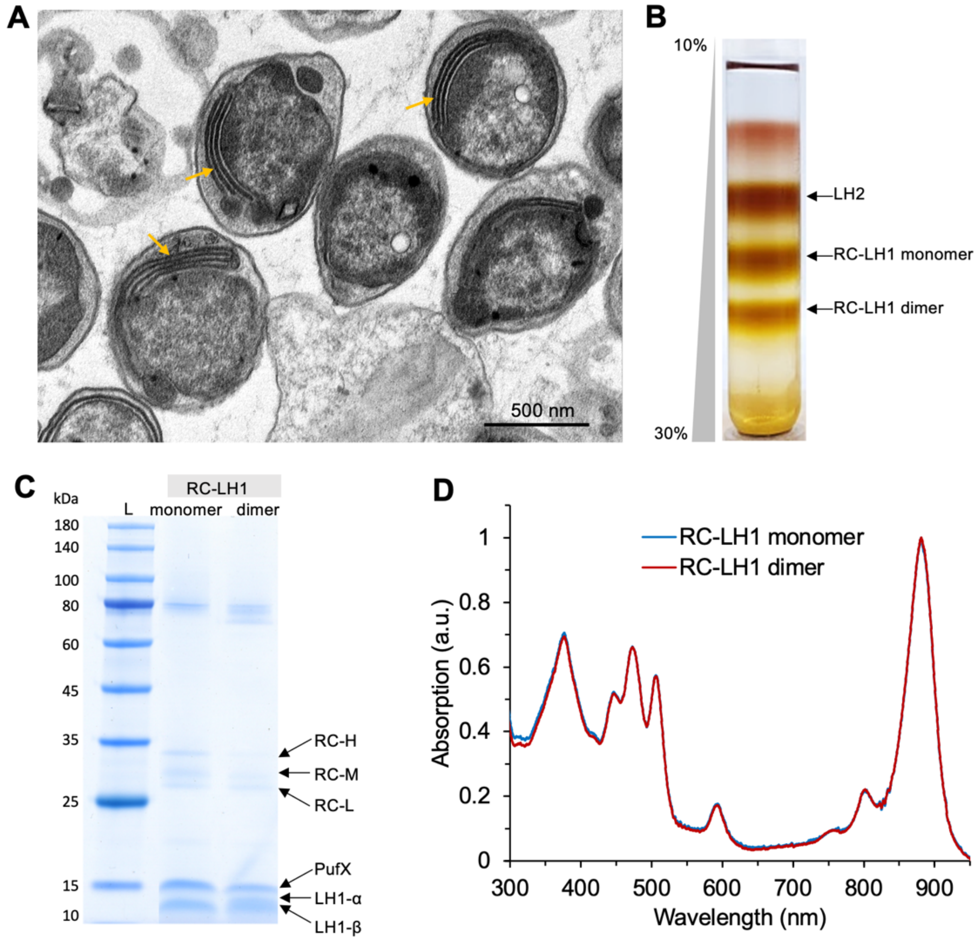

Fig. S1. Isolation and characterization of the *Rba. blasticus* RC−LH1 core complexes. (A) Thin-section electron microscopy images of *Rba. blasticus*. (B) Separation of membrane proteins from *Rba. blasticus* using a 10-30 % continuous sucrose gradient. The pigmented fractions were identified as LH2, RC–LH1 monomer and RC–LH1 dimer from the top to the bottom. (C) SDS-PAGE of purified RC–LH1 monomers and dimers, stained by Coomassie Brilliant Blue. (D) Absorption spectra of purified *Rba. blasticus* RC–LH1 complexes.

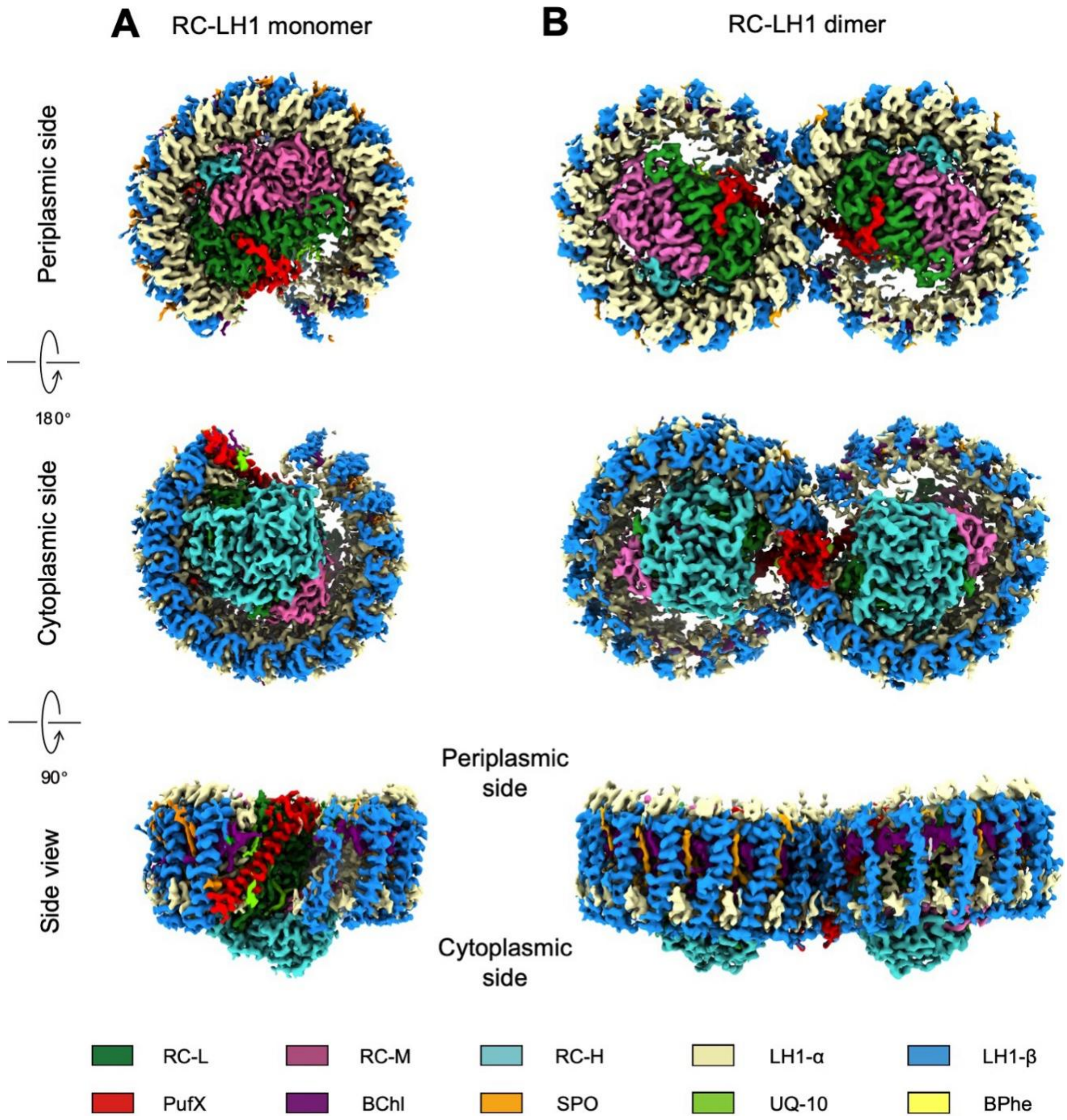

Fig. S2. Color-coded electron density maps of the RC–LH1 core complexes from *Rba. blasticus*. (A) RC–LH1 monomer. (B) RC–LH1 dimer. Color scheme is presented as the same as shown in Fig. 1: LH1-α, wheat; LH1-β, light blue; PufX, red; RC-L, green; RC-M, magenta; RC-H, light sea green; BChls, purple; BPhes, yellow; carotenoids, orange; quinones, lawn green.

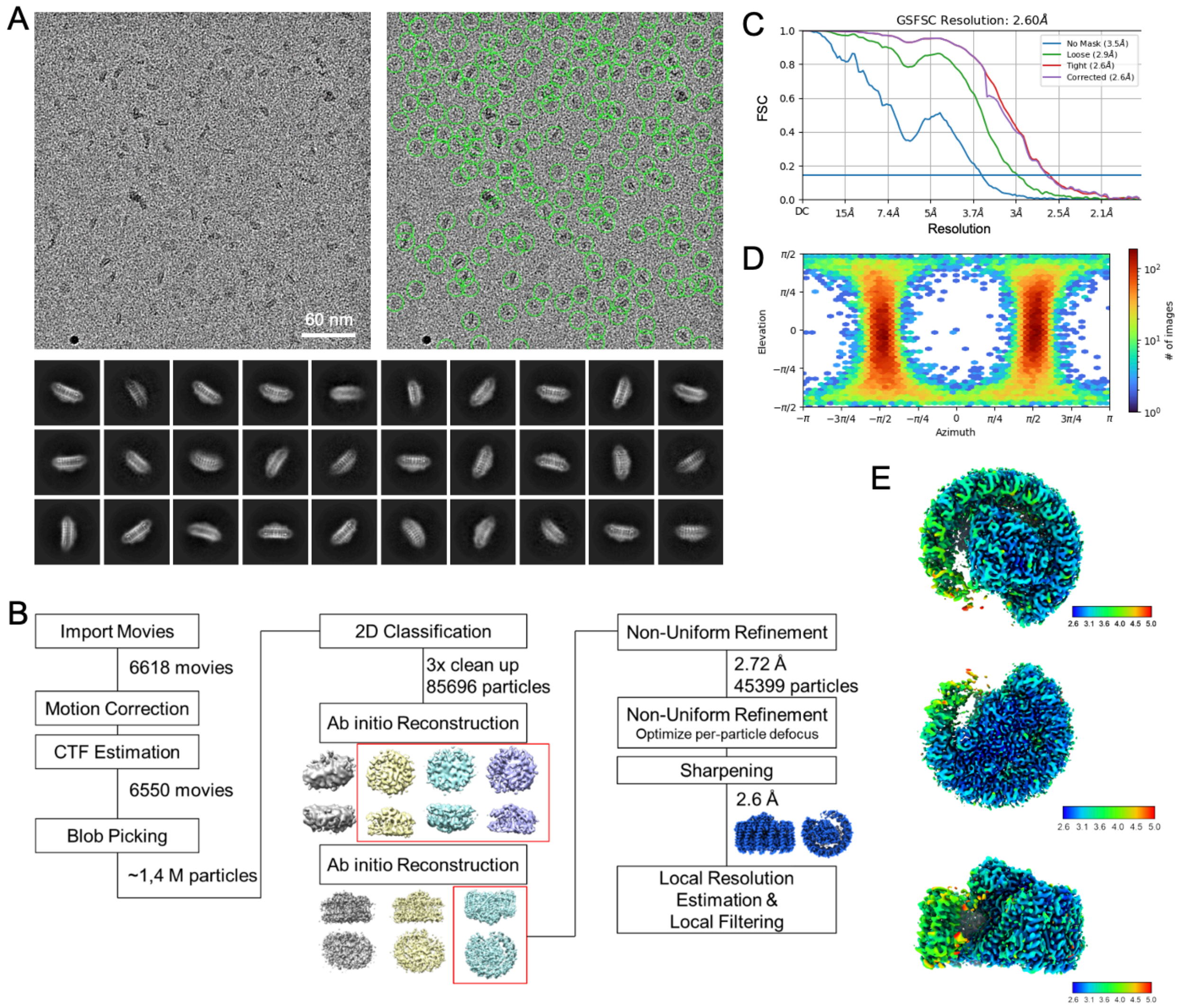

Fig. S3. Cryo-EM data process of the *Rba. blasticus* RC–LH1 monomer. (A) Motioncorrected example of a cryo-EM captured movie is shown on top left and all particles picked from that micrograph are marked with green circles shown on top right. Imaged area is 345.2 nm^2. The diameter of the green circles measures 196 Å. Representative reference-free 2D class averages are shown at the bottom. (B) Overview of cryo-EM data processing workflow for the monomer dataset. Selected 3D class that went into further processing is marked with a red rectangle. (C) Fourier Shell Correlation (FSC) curves generated by cryoSPARC. Global resolution values were calculated according to the gold-standard FSC = 0.143. (D) Angular distribution calculated in cryoSPARC for particle projections. Heat map shows number of particles for each viewing angle. (E) Local resolution of the cryo-EM map as seen from the side view (top left and top right), periplasmic view (bottom left), and cytoplasmic view (bottom right), estimated by cryoSPARC.

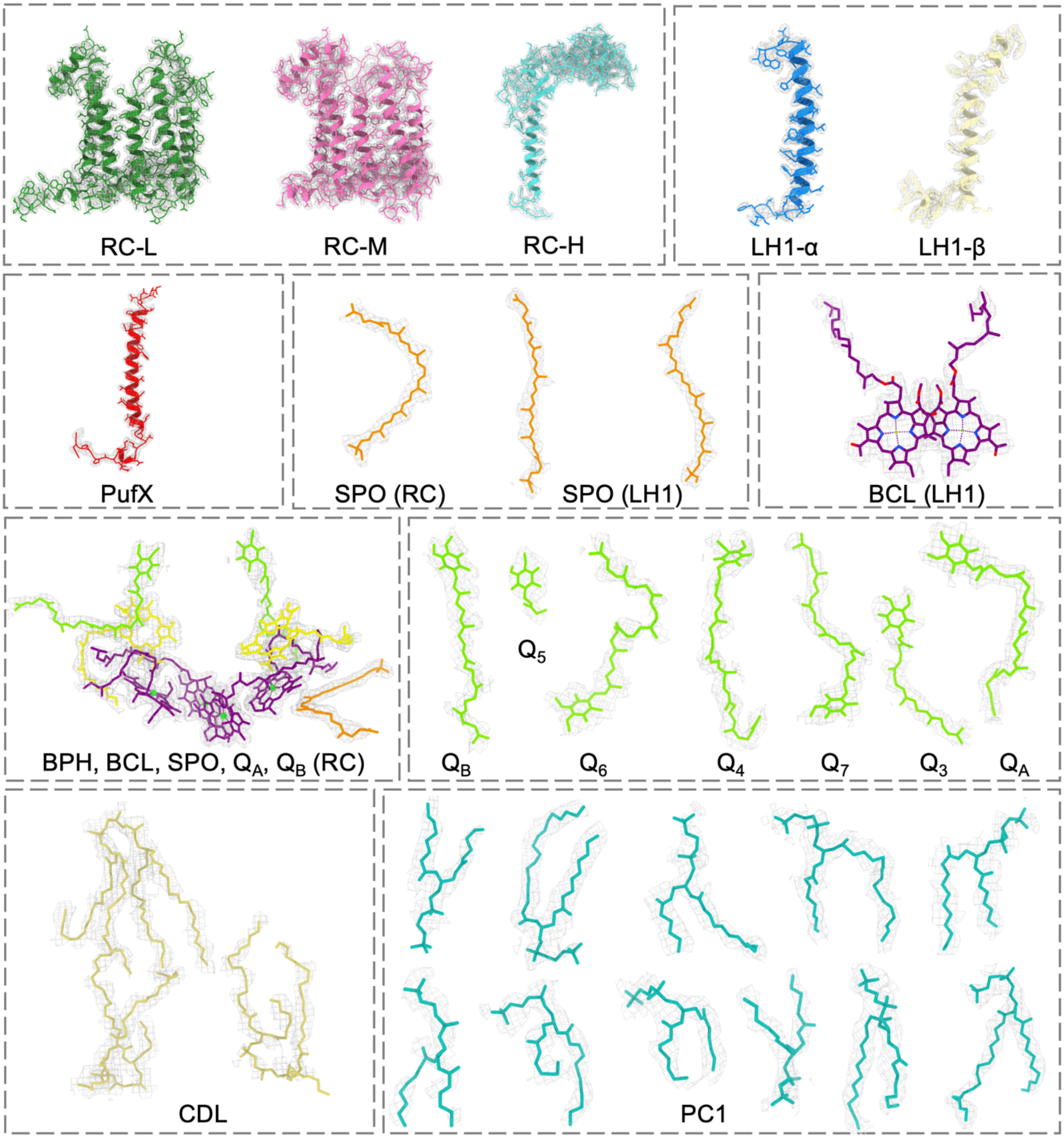

Fig. S4. Cryo-EM map densities and structural models of protein peptides and cofactors in the RC–LH1 monomer. Based on the low-resolution local densities, PufX was only built starting from the 14th amino acid at the N-terminus, without the first 13 amino acids.

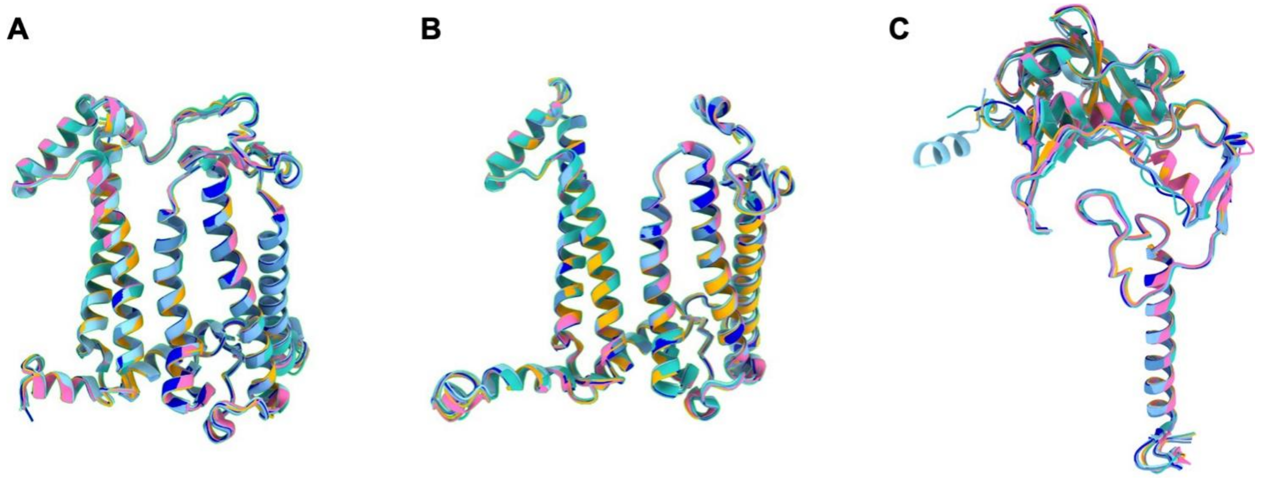

Fig. S5. Comparison of the RC-M (A), RC-L (B), and RC-H (C) subunits of the RC–LH1 monomers from *Rba. blasticus*, *Rba. capsulatus* (PDB ID: 8B64), *Rba. veldkampii* (PDB ID: 7DDQ), and *Rba. sphaeroides* (PDB ID: 7VNY, 7PIL, 7VOR, 7F0L). The *Rba. blasticus* model is colored in orange, the *Rba. veldkampii* model is colored in light sea green, the *Rba. capsulatus* model is colored in hot pink, and the *Rba. sphaeroides* models are colored in light blue (PDB ID: 7VNY), dark turquoise (PDB ID: 7PIL), sky blue (PDB ID: 7VOR) and steel blue (PDB ID: 7F0L).

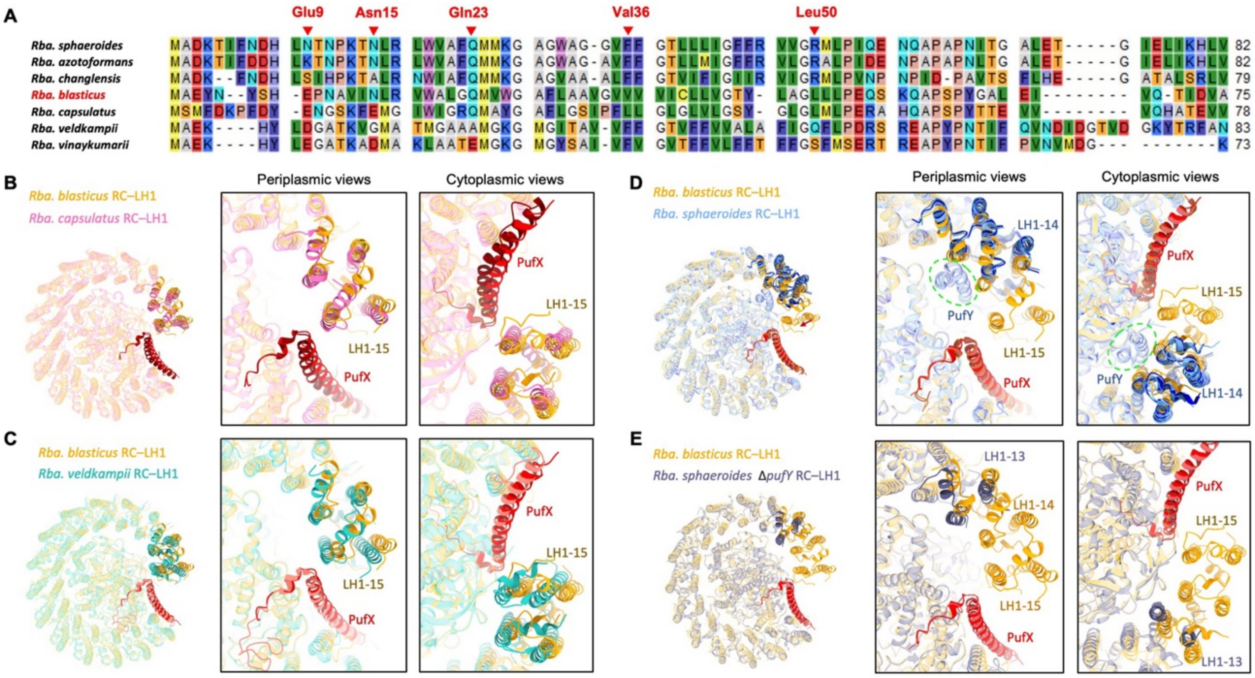

Fig. S6. Comparison of the PufX-containing RC–LH1 monomers. (A) Multiple sequence alignment of PufX. (B-E) Comparison of the structures of RC–LH1 monomers from *Rba. blasticus*, *Rba. capsulatus* (PDB ID: 8B64*), Rba. veldkampii* (PDB ID: 7DDQ), *Rba. sphaeroides* (PDB ID: 7VNY, 7PIL, 7F0L) and *Rba. sphaeroides* Δ*pufY* (PDB ID: 7VNM). The 15th LH1 subunit is found in the RC–LH1 monomers from *Rba. blasticus*, *Rba. veldkampii*, and *Rba. capsulatus*, but is absent in WT *Rba. sphaeroides* RC–LH1 monomer. Furthermore, *Rba. sphaeroides* Δ*pufY* lacks both the 14th and 15th LH1 subunits. *Rba. blasticus* model is colored in orange, with PufX colored in red. *Rba. capsulatus* model is colored in hot pink, with PufX colored in dark red. *Rba. veldkampii* model is colored in light sea green, with PufX colored in salmon. *Rba. sphaeroides* models are colored in light blue (PDB ID: 7VNY), dark turquoise (PDB ID: 7PIL), and steel blue (PDB ID: 7F0L) with PufXs colored in red, sienna, and chocolate. *Rba. sphaeroides* Δ*pufY* model is colored in dark slate blue with PufX colored in red. The RC–LH1 monomer of *Rba. sphaeroides* contains an additional PufY subunit (dashed circle).

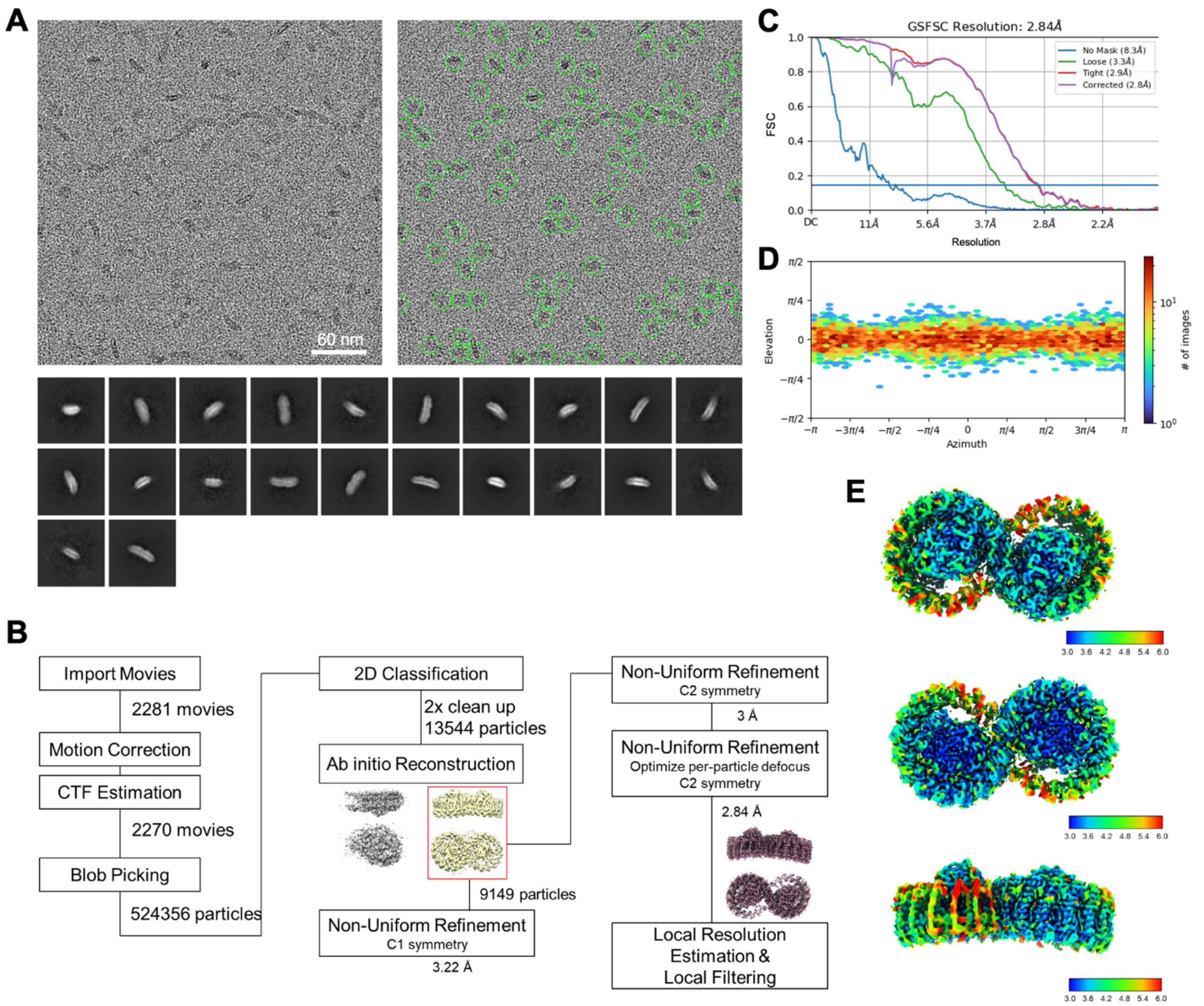

Fig. S7. Cryo-EM data process of the *Rba. blasticus* RC–LH1 dimer. (A) Motioncorrected example of a cryo-EM captured movie is shown on top left and all particles picked from that micrograph are marked with green circles shown on top right. Imaged area is 345.2 nm2. The diameter of the green circles measures 270 Å. Representative reference-free 2D class averages are shown at the bottom. (B) Overview of cryo-EM data processing workflow for the monomer dataset. Selected 3D class that went into further processing is marked with a red rectangle. (C) FSC curves generated by cryoSPARC. Global resolution values were calculated according to the gold-standard FSC = 0.143. (D) Angular distribution calculated in cryoSPARC for particle projections. Heat map shows number of particles for each viewing angle (less = blue, more = red). (E) Local resolution of the cryo-EM map as seen from the side view (top left and top right), periplasmic view (bottom left), and cytoplasmic view (bottom right), estimated by cryoSPARC.

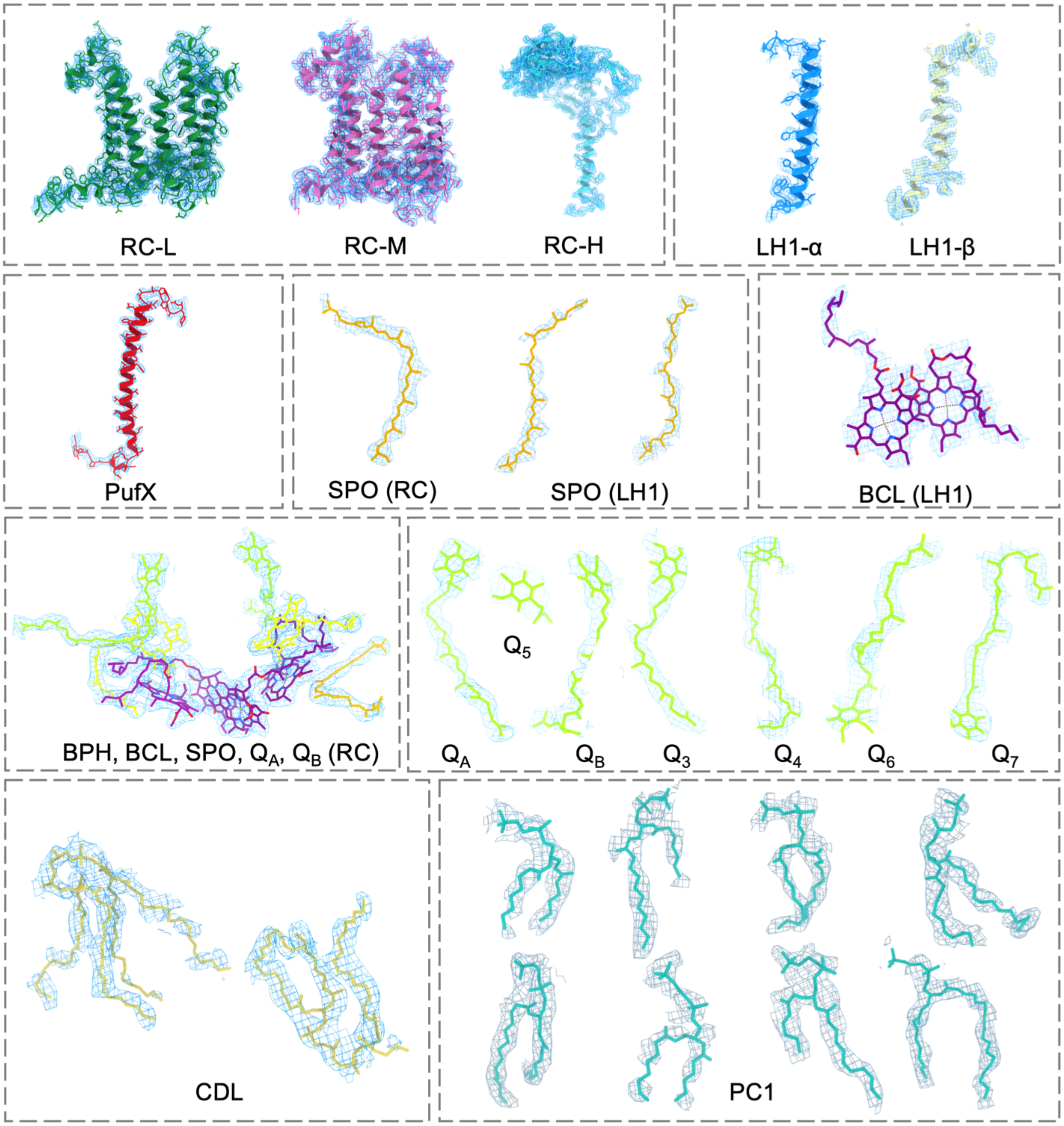

Fig. S8. Cryo-EM map densities and structural models of protein peptides and cofactors in the RC–LH1 dimer. Based on the local densities, PufX was built starting from the 5th amino acid at the N-terminus.

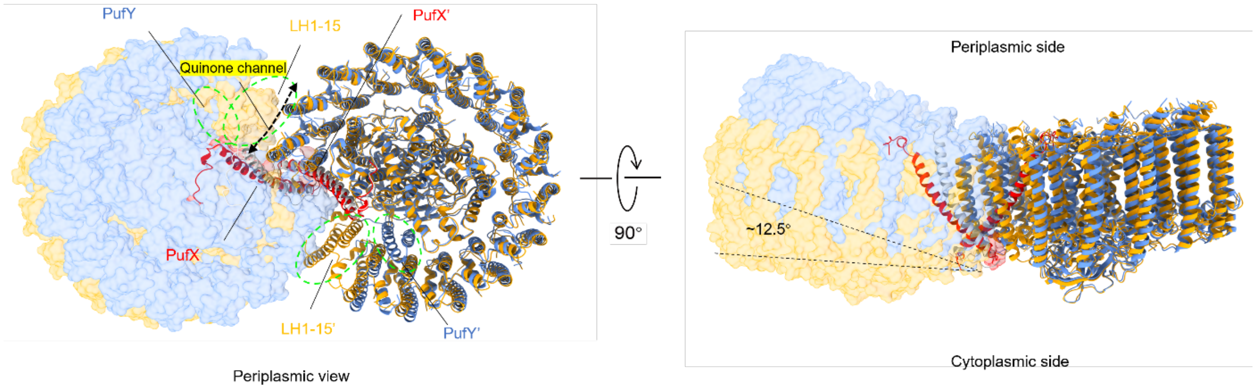

Fig. S9. Comparison of the RC–LH1 dimers from *Rba. blasticus* and *Rba. sphaeroides*. The overall structures of the reported *Rba. sphaeroides* RC–LH1 dimers (PDB ID: 7VOR, 7PQD, 7VY2) exhibit similarities. Therefore, we selected the structure with PDB ID: 7VOR as the representative for conducting the comparative analysis. The *Rba. blasticus* model is colored in orange, with PufX colored in red. The *Rba. sphaeroides* models are colored in light blue, with PufXs colored in grey. Monomers on the right of each model were superimposed, and the other monomer followed in its relation to the first. The 15th and 15th' LH1 subunits of the RC–LH1 dimer from *Rba. blasticus* and the PufY subunits of the RC–LH1 dimer from *Rba. sphaeroides* are annotated with dashed circles.

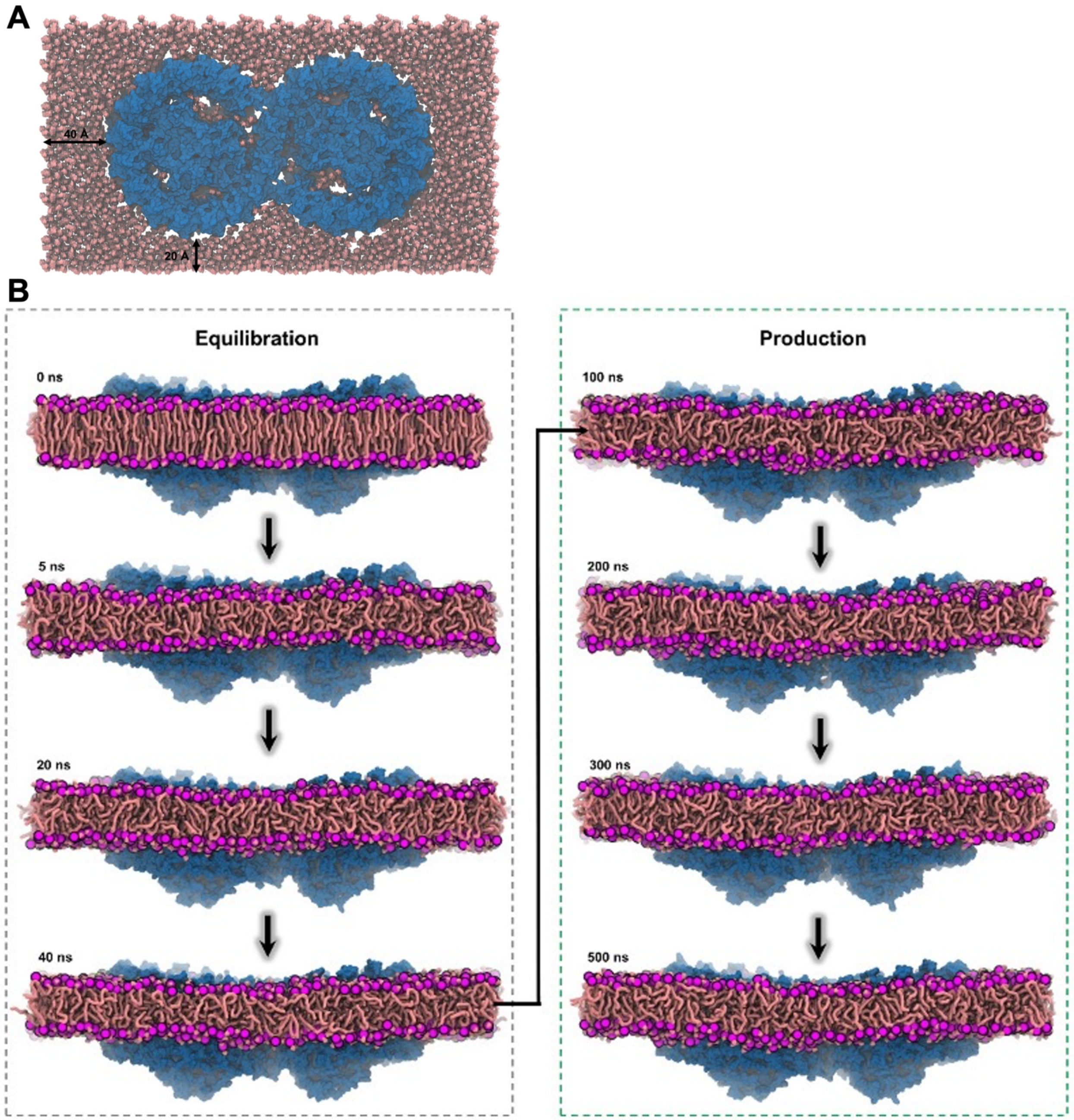

Fig. S10. Computational simulations of dimer-induced membrane curvature. (A) Top view of the structural model of *Rba. blasticus* RC–LH1 dimer embedded in a phospholipid bilayer. Given that the impact of RC–LH1 dimerization on the lipid bilayer is mainly along the long axis, we extended the phospholipid membrane by at least 40 Å in the direction of the protein dimer's long axis, and by 20 Å in the short axis direction. (B) Side views of the structures of the RC–LH1 dimer in the lipid bilayer at 0, 20, and 40 ns of the equilibration and at 500 ns of the production all-atom molecular dynamics (AAMD) simulations are shown. Equilibration 0 ns to 40 ns represents the membrane curvature induced by the bent conformation of the RC–LH1 dimer (left). The curved structure of the lipid bilayer was maintained during the 500-ns unrestrained AAMD simulations. RC–LH1 dimers are shown with molecular surfaces colored in dark blue.

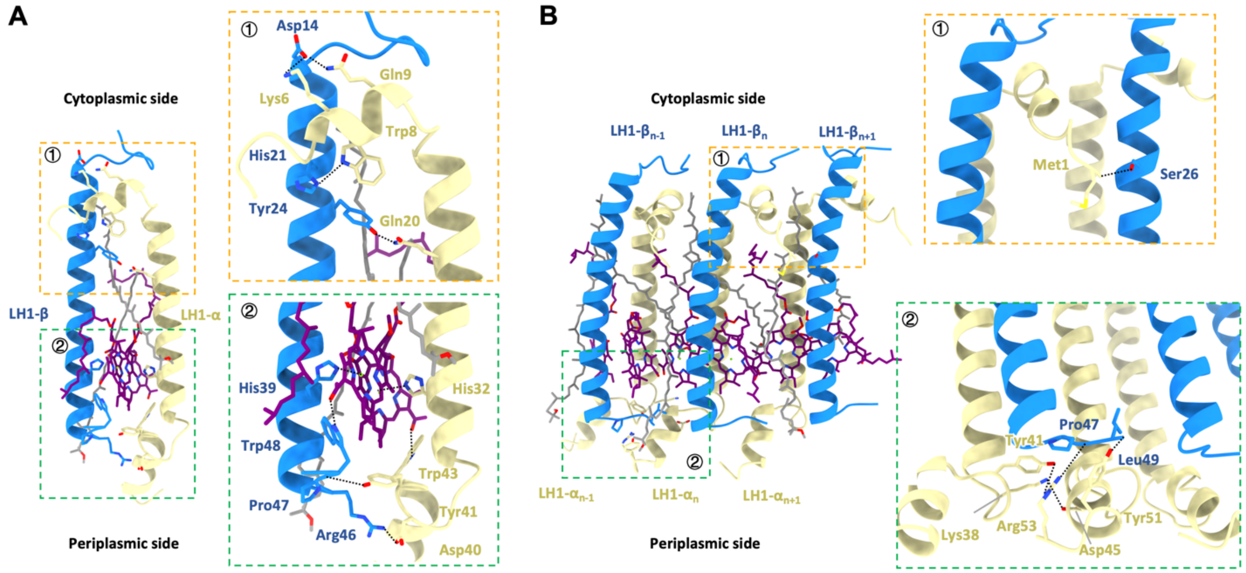

Figure S11. Interactions within the LH1 subunit and between neighboring LH1 subunits. (A) Representative LH1 subunit with interacting residues depicted as sticks. Close-up views of the interactions are displayed in boxes on the side. (B) Representative LH1-LH1 interface with interacting residues shown as sticks. Close-up views of the interactions between neighboring LH1 subunits are presented in boxes on the side. BChls are colored in purple and SPOs are colored in grey.

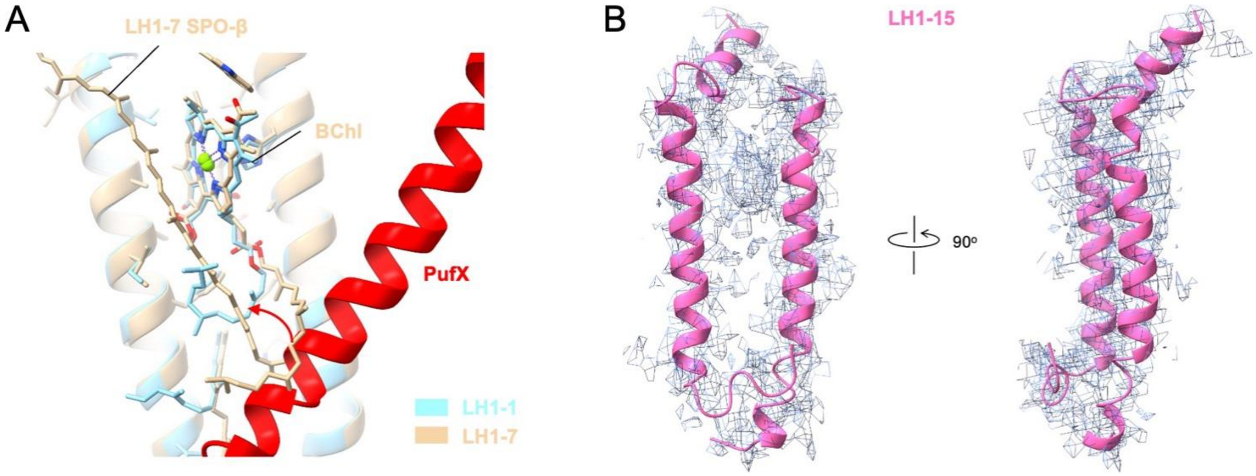

Fig. S12. The LH1-1 and LH1-15 subunits of *Rba. Blasticus*. (A) Differences in SPOs and BChls binding in LH1 and LH7 subunits of *Rba. blasticus* RC–LH1. (B) The poor electron density of the LH1-15 subunit.

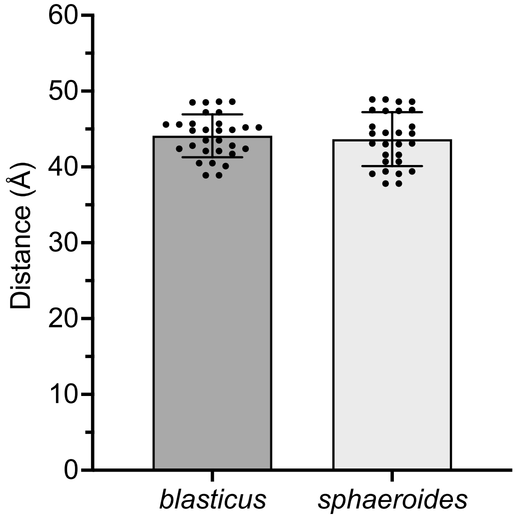

Fig. S13. Mg-Mg distances from LH1-associated BChls to the special pair of BChls in the RC of *Rba. blasticus* RC–LH1–PufX, compared to *Rba. sphaeroides* RC–LH1–PufX–PufY. p = 0.6016, indicating no significant difference.

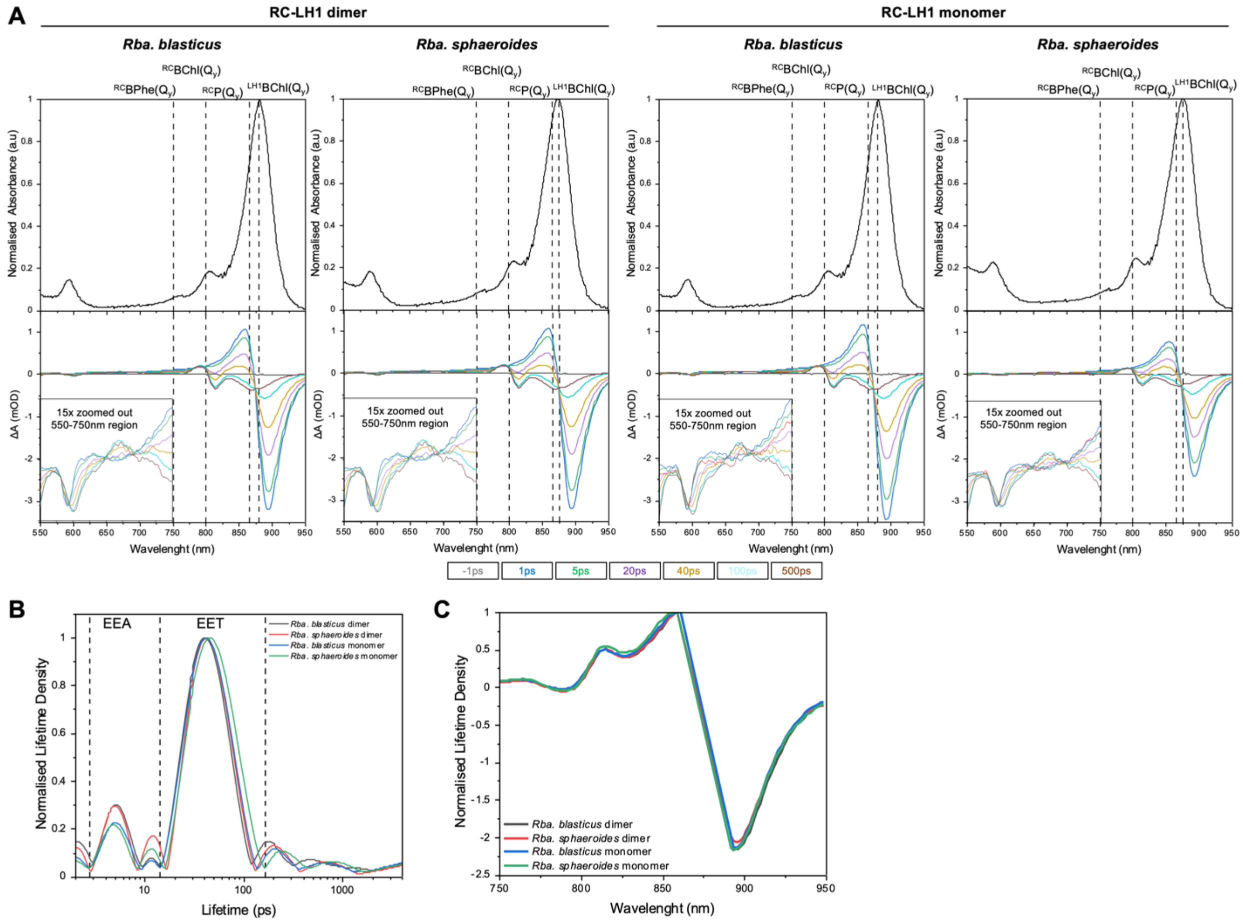

Fig. S14. Transient absorption (TA) spectra, and Combined Lifetime Density Kinetic Traces (LDKT) and Lifetime Averages Difference Spectra (LADS) of the RC–LH1 complexes. (A) TA spectra in both visible (550-750 nm) and near-infrared (750-950nm) regions of RC–LH1 complexes. Inset shows the zoomed-in visible regions. (B) Lifetime density kinetic traces associated with the kinetic process, obtained for all *Rba. blasticus* RC–LH1 and *Rba. sphaeroides* RC–LH1 and supercomplexes. (C) The lifetime averaged difference spectra (LADS) showing the wavelength dependent average pre-exponential factor of lifetimes on the two dominating processes. LADS associated with the most dominating process at ~40 ps show energy transfer from BChls in LH1 (^{LH1}BChls) to the special pair of BChls in the reaction centre through excitation energy transfer (EET). This is evident from the derivative line shape band at ~800 nm assigned to perturbation of ^{RC}BChl *a* and the loss of ^{LH1}BChl *a* ground state bleach and stimulated emission at wavelengths longer than 875 nm, and (^{LH1}BChl *a*)* photoinduced absorption at ~865 nm.

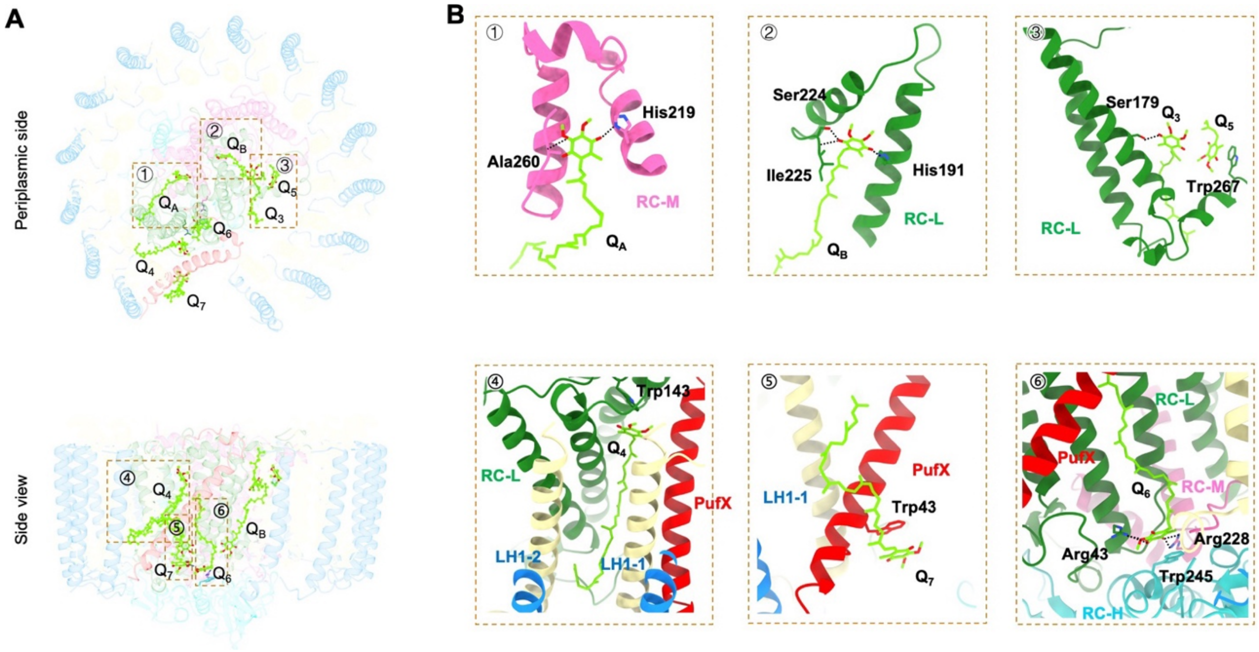

Fig. S15. Quinones in the *Rba. blasticus* RC–LH1 complexes. (A) Positions of identified quinone molecules in RC–LH1 monomer. (B) Close-up views of identified quinones. Interacting residues are marked and shown in sticks. UQ-10 molecules are coloured in lawn green.

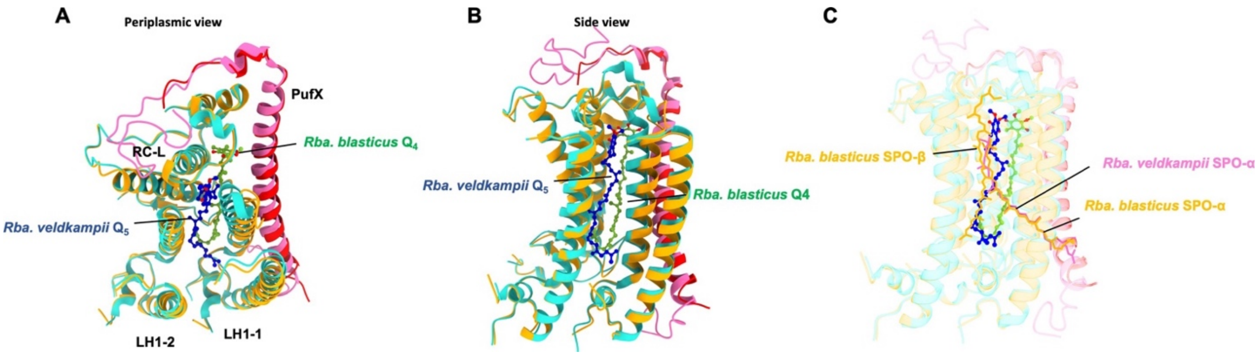

Fig. S16. Comparison of the structures of *Rba. blasticus* RC–LH1 and *Rba. veldkampii* RC–LH1 (PDB ID: 7DDQ) near *Rba. blasticus* Q_4. (A) Periplasmic view of the structural comparison. (B) Side view of the structural comparison in the membrane plane. (C) Side view of the carotenoids. The RC-L, LH1-1, and LH1-2 subunits of *Rba. veldkampii* RC–LH1 are colored in light sea green. The RC-L, LH1-1, and LH1-2 subunits of *Rba. blasticus* RC–LH1 are colored in orange.

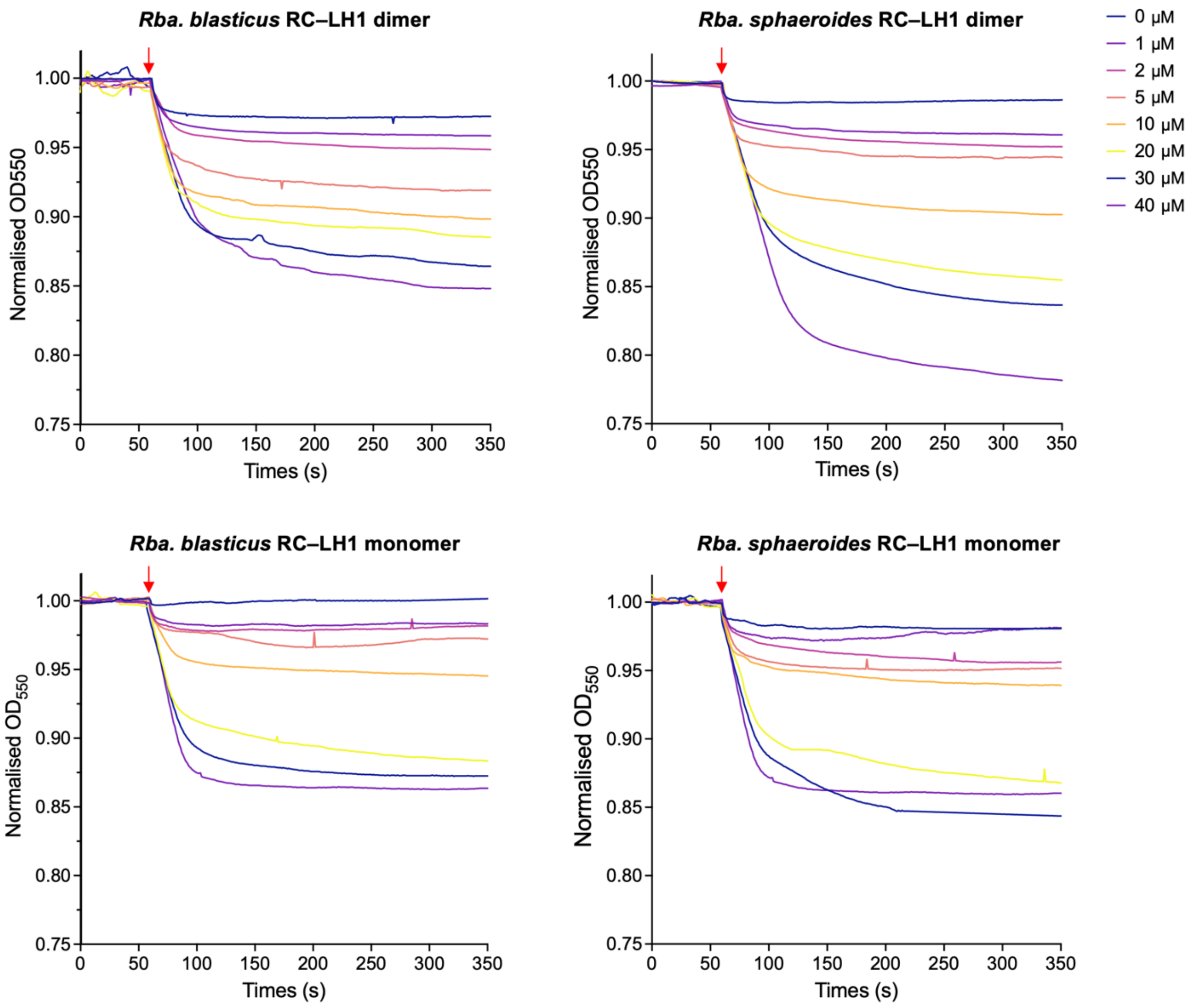

Fig. S17. Absorbance changes at 550 nm upon illumination of *Rba. blasticus* RC–LH1 dimers and monomers (left), in comparison with *Rba. sphaeroides* RC–LH1 dimers and monomers. Assays were carried out with the RC–LH1 concentration of 30 nM of *Rba. blasticus* and *Rba. sphaeroides* RC–LH1 dimers and monomers. The RC–LH1 complexes were incubated with 30 μM reduced cytochrome c_2 and various concentrations of UQ_2, as indicated, at 4 °C overnight. Red arrows indicate the beginning of the illumination period.

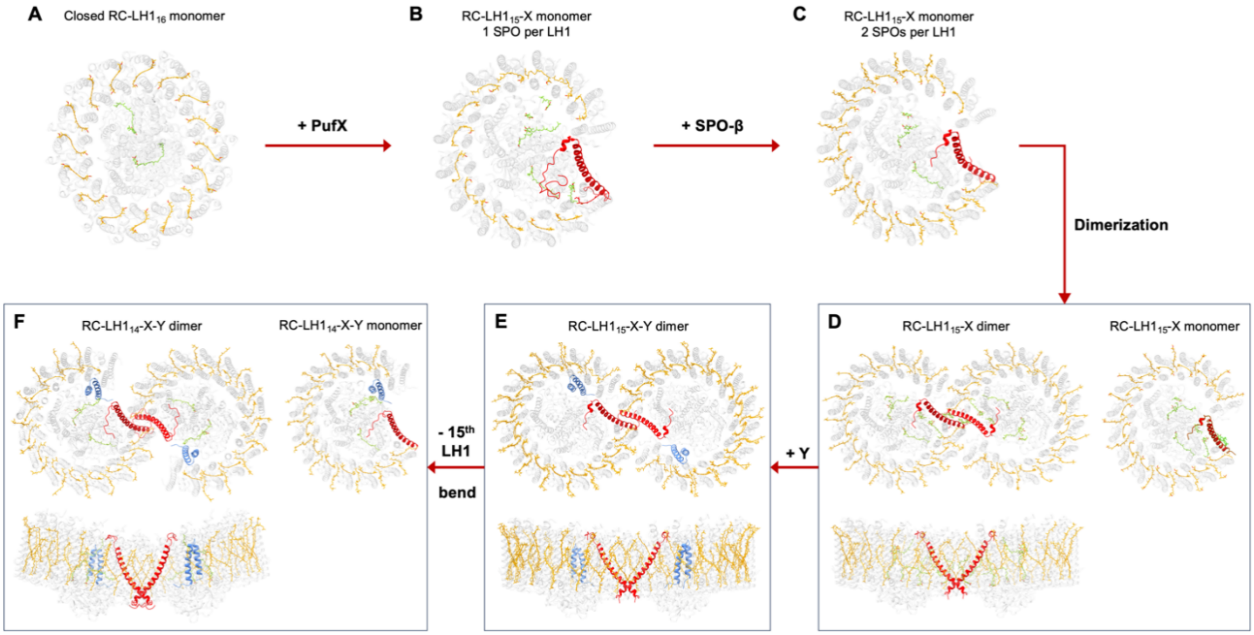

Fig. S18. Structural variations of *Rhodobacter* RC–LH1 complexes. (A) RC–LH1$_{15}$ monomer from *Tch. tepidum* (PDB ID: 3WMM), showing a closed LH1 ring. (B) RC–LH1$_{15}$–PufX monomer from *Rba. veldkampii* (PDB ID: 7DDQ), with an open in the LH1 ring and one SPO per LH1 subunit. (C) RC–LH1$_{15}$–PufX monomer from *Rba. capsulatus* (PDB ID: 8B64), with an open in the LH1 ring and two SPOs per LH1 subunit. (D) RC–LH1$_{15}$–PufX dimer and monomer from *Rba. blasticus*, containing two SPOs per LH1 subunit. (E) RC–LH1$_{15}$–PufX–PufY dimer from *Rca. bogoriensis*, The dimer structure was built based on *Rba. blasticus* and *Rba. sphaeroides* RC–LH1 dimer structures, as the structural file of *Rca. bogoriensis* RC–LH1 dimer has not been available in PDB. The *Rca. bogoriensis* RC–LH1 monomer structure has not been reported. (F) RC–LH1$_{14}$–PufX–PufY monomer from *Rba. sphaeroides* (monomer, PDB ID: 7VNY; dimer, PDB ID: 7VOR). PufX and PufY subunits are colored in red and blue, respectively. Quinones are colored in lawn green. Carotenoids on the LH1 ring are colored in orange.

Table S1. Cryo-EM data collection, refinement and validation statistics.

	**RC–LH1 Monomer (PDB-8YGL) (EMDB-39255)**	**RC–LH1 Dimer (PDB-8YGD) (EMDB-39244)**	**RC–LH1 Dimer**
Data collection and processing			
Magnification	130,000 x	130,000 x	130,000 x
Voltage (kV)	300	300	300
Electron exposure (e^{-}/Å^2)	40	40	40
Defocus range (μm)	0,8-2.0	0,8-2.0	0.8-2.0
Pixel size (Å)	0.929	0.929	0.929
Symmetry imposed	C1	C2	C1
Initial particle images (no.)	1,476,836	524,356	524,356
Final particle images (no.)	45399	9149	9149
Map resolution (Å) FSC: 0.143	2.6	2.84	3.22
Refinement			
Initial model used (PDB code)	7VNY	7VNY	
Model resolution (Å)	2.79	2.79	
FSC threshold	0.143	0.143	
Model composition			
Non-hydrogen atoms	23222	46510	
Protein residues	2297	4602	
B factors (Å2)			
Protein	26.24	89.46	
Ligend	25.02	89.65	
R.m.s. deviations			
Bond lengths (Å)	0.007	0.014	
Bond angles (°)	0.770	1.451	
Validation			
MolProbity score	1.71	2.13	
Poor rotamers (%)	0.00	1.6	
Ramachandran plot			
Favored (%)	98.61	95.97	
Allowed (%)	1.39	4.03	
Disallowed (%)	0.00	0.00	
